# Surface Treatment With Hydrophobic Coating Reagents (Organosilanes) Strongly Reduces the Bioactivity of Synthetic Amorphous Silica *in vitro*

**DOI:** 10.3389/fpubh.2022.902799

**Published:** 2022-06-21

**Authors:** Martin Wiemann, Antje Vennemann, Tobias B. Schuster, Jürgen Nolde, Nils Krueger

**Affiliations:** ^1^IBE R&D Institute for Lung Health gGmbH, Münster, Germany; ^2^Evonik Operations GmbH, Hanau-Wolfgang, Germany; ^3^Grace Europe Holding GmbH, Worms, Germany

**Keywords:** synthetic amorphous silica, surface treatment, hydrophobicity, organosilanes, siloxanes, alveolar macrophage

## Abstract

Synthetic amorphous silica (SAS) is industrially relevant material whose bioactivity *in vitro* is strongly diminished, for example, by protein binding to the particle surface. Here, we investigated the *in vitro* bioactivity of fourteen SAS (pyrogenic, precipitated, or colloidal), nine of which were surface-treated with organosilanes, using alveolar macrophages as a highly sensitive test system. Dispersion of the hydrophobic SAS required pre-wetting with ethanol and extensive ultrasonic treatment in the presence of 0.05% BSA (Protocol 1). Hydrophilic SAS was suspended by moderate ultrasonic treatment (Protocol 2) and also by Protocol 1. The suspensions were administered to NR8383 alveolar macrophages under serum-free conditions for 16 h, and the release of LDH, GLU, H_2_O_2_, and TNFα was measured in cell culture supernatants. While seven surface-treated hydrophobic SAS exhibited virtually no bioactivity, two materials (AEROSIL® R 504 and AEROSIL® R 816) had minimal effects on NR8383 cells. In contrast, non-treated SAS elicited considerable increases in LDH, GLU, and TNFα, while the release of H_2_O_2_ was low except for CAB-O-SIL® S17D Fumed Silica. Dispersing hydrophilic SAS with Protocol 1 gradually reduced the bioactivity but did not abolish it. The results show that hydrophobic coating reagents, which bind covalently to the SAS surface, abrogate the bioactivity of SAS even under serum-free *in vitro* conditions. The results may have implications for the hazard assessment of hydrophobic surface-treated SAS in the lung.

## Introduction

**Figure 1:**
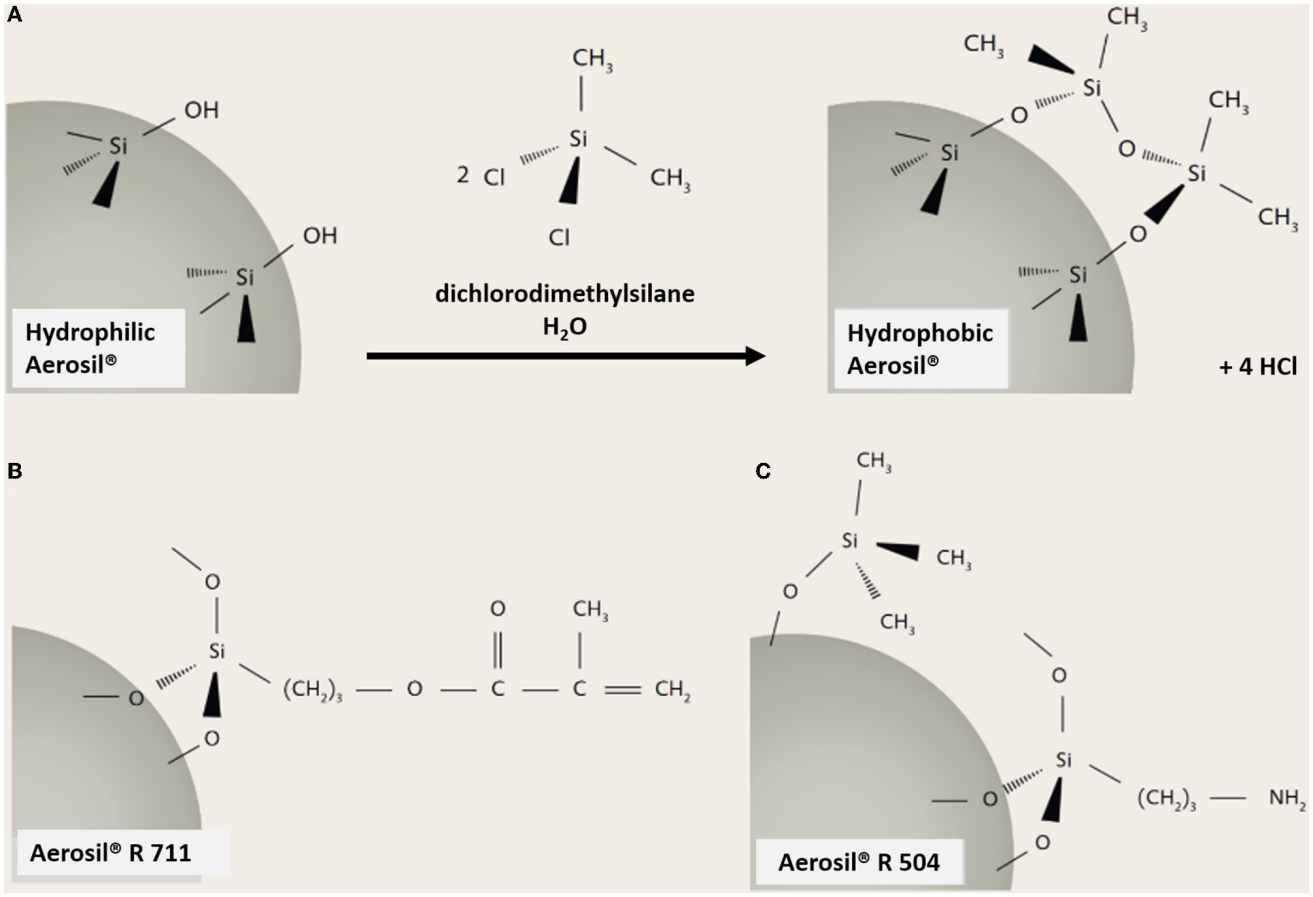
Reaction of organosilanes with silanol groups of SAS and typical reaction products. **(A)** Reaction of dichlorodimethylsilane (DDS) with a hydrophilic Aerosil® generates a hydrophobic AEROSIL® **(B)** methacrylate functionalities at the surface of AEROSIL® R 711; **(C)** partly aminated surface of AEROSIL® R 504. All parts of this figure are taken from “Evonik Industries. AEROSIL®—Fumed Silica, Technical Overview” (8).

A large variety (precipitated, pyrogenic, silica gel or colloidal forms) of synthetic amorphous silica (SAS) is produced and used for many industrial applications. SAS is incorporated in consumer products, cosmetics, feed, pharmaceuticals, or food (1–4) and serves as thickeners, fillers, flow enhancing agents, or stabilizers (1, 3, 5–7). However, several of these applications are incompatible with the hydrophilic SiO_2_ surface. Chemical modifications are in use to render the SAS particle surface hydrophobicity, thus allowing their incorporation, for example, into polymers, such as silicone rubber, non-water-based paint and coating formulations, toner products, adhesives and sealants, cable compounds, and resin systems. A versatile and widespread industrial process to achieve the hydrophobicity of SAS is the surface treatment with organosilanes as shown in Figure 1. A multitude of different hydrophobic moieties is available, enabling inventors to design and adapt the properties of SAS (such as polarity) to specific material requirements. Organosilanes are chemically bound through SiO_2_ (Figure 1) or form other types of bonds as outlined in Table 1. Silanol groups are suspected to be mainly responsible for the biological activity of SAS (9). A chemical modification or capping of these groups may, therefore, lead to a markedly reduced bioactivity, which should be demonstrable *in vitro*. However, the effects of SAS surface treated with organosilanes have not yet been systematically investigated with sensitive *in vitro* test systems.

**Table 1:** List of materials, coating agents, surface modifications, and properties of SAS.

**Substance name**	**Source**	**Type**	**Treating agent, abbreviation (CAS No.)**	**Chemical name (CAS No.)**	**Primary particle size (TEM)**	**Aggregate size (TEM)**	**Skeletal density (g/ml)**	**BET (m^**2**^/g)**	**Further properties/purity**
LUDOX® SM	GRACE	CS	None	112926-00-8 (ex 7631-86-9)	7 nm	No aggregates	2.2	320–400	30% in H2O; negatively particle charge stabilized with Na+
LUDOX® TM-50	GRACE	CS	None	112926-00-8 (ex 7631-86-9)	22 nm	No aggregates	2.2	110–150	50% in H2O; negatively particle charge stabilized with Na+
CAB-O-SIL® S17D	CABOT	FS	None	112945-52-5 (ex 7631-86-9)	9 nm^1^	D50: 66 nm, D10: 31 nm, D90: 123 nm	2.3	390–430	Non-porous
CAB-O-SIL (E)L-90	CABOT	FS	None	112945-52-5 (ex 7631-86-9)	16 nm^1^	D50: 143 nm, D10: 60 nm, D90: 278 nm	2.3	83–97	
AEROSIL® 50	EVONIK	FS	None	112945-52-5 (ex 7631-86-9)	25 nm^1^	Feret min, D50: 263 nm	2.3	35–65	SiO2 content (based on ignited material): ≥99.8%
AEROSIL® R816	EVONIK	FS	Trimethoxyhexadecyl-silan (199876-45-4)	Silane; hexadecyltrimethoxy-, hydrolysis products with silica (199876-45-4)	8 nm^1^	Feret min, D50: 121 nm	1.9–2.5	170–210	SiO2 content (based on ignited material): ≥99.8%, Carbon content 0.9–1.8%
CAB-O-SIL® TGC413TRD	CABOT	dCS	Hexamethyldisilazane HDMZ (999-97-3)	Silanamine; 1,1,1-trimethyl-N-(trimethylsilyl)-, hydrolysis products with silica (68909-20-6)	50 nm	No aggregates	2.2–2.3	45–70	Non-porous, colloidal silica [(trimethylsilyl)oxy]-modified
CAB-O-SIL®TS610	CABOT	FS	Dichlorodimethylsilane DMS (75-78-5)	Silane; dichlorodimethyl-, reaction products with silica (Silica-[(dimethylsilyl)oxy]-modified) (68611-44-9)	12 nm^1^	D50: 106 nm, D10: 49 nm, D90: 203 nm	2.2	105–145	Non-porous, silane, dichlorodimethyl-, reaction products with silica
CAB-O-SIL® TS720	CABOT	FS	Polydimethylsiloxane, PDMS (63148-62-9)	Silicones and siloxanes, dimethyl-, reaction products with silica ((67762-90-7)	12 nm^1^	D50: 122 nm, D10: 52 nm, D90: 215 nm	1.9	105–135	Non-porous, siloxanes and silicones, di-Me, reaction products with silica
HDK® H15	WACKER	FS	Dichlorodimethylsilane DMS (75-78-5)	Silane; dichlorodimethyl-, reaction products with silica (Silica-[(dimethylsilyl)oxy]-modified) (68611-44-9)	15 mn^1^	Number-based D50: 248 nm, D10: 43 nm, D90: 554 nm.	2.2	150^2^	SiO2 content (based on ignited material): ≥99.8%
HDK® H2000	WACKER	FS	Hexamethyldisilazane, HDMZ (999-97-3)	Silanamine; 1,1,1-trimethyl-N-(trimethylsilyl)-, hydrolysis products with silica (68909-20-6)	12 nm^1^	Number:-based D50: 63 nm, D10: 24 nm, D90: 160 nm	2.2	200^2^	SiO2 content (based on ignited material): ≥99.8%
AEROSIL® R504	EVONIK	FS	3-Aminopropyl-triethoxysilane, AMEO (919-30-2) plus Hexamethyldisilazane, HDMZ (999-97-3)	Silanamine; 1,1,1-trimethyl-N-(trimethylsilyl)-, hydrolysis products with silica and 3-(triethoxysilyl)-1-propanamine (199876-44-3)	n.m.	n.m.	2.2	125-175	SiO2 content (based on ignited material): ≥99.8%, Carbon content: ca. 2.0–4.5%; Trimethylsilyl and Aminopropylsilyl groups
AEROSIL® R711	EVONIK	FS	Trimethoxysilyl propylmethacrylate, MEMO (2530-85-0)	2-Propenoic acid; 2-methyl-, 3-(trimethoxysilyl)propylester, reaction products with silica (100402-78-6)	8 nm^1^	Feret min, D50: 123 nm	1.9–2.5	125–175	SiO2 content (based on ignited material): ≥99.8%, Carbon content: ca. 4.5–6.5%; Methacrylsilyl groups
SIPERNAT® D17	EVONIK	PS	Polydimethylsiloxane PDMS (63148-62-9)	Silicones and siloxanes, dimethyl-, reaction products with silica (67762-90-7)	15 nm^1^	Feret min, D50: 89 nm	2.0	100	Dimethylsilyl groups, Carbon content: ca. 1.7%

*FS, fumed (pyrogenic) silica; CS, colloidal silica; dCS, dried colloidal silica; PS, precipitated silica. ^1^ Constituent particle size (internal structures which cannot be isolated, D50, number-based by TEM). ^2^ Prior to coating*.

The choice of an *in vitro* testing system should reflect the relevant route of particle uptake. In case of pyrogenic (“fumed”), precipitated, or dried colloidal surface-treated SAS, which are distributed as dry powder nanostructured materials, unintentional inhalation needs to be considered as a possible way of particle uptake into the body (10, 11) and the appropriate risk assessment should focus on possible effects of SAS in the lung. SAS, especially without surface-functionalization, is known to induce transient and gradually more pronounced lung inflammation in rodents (10, 12–17). In line with this, also *in vitro* studies carried out with various cell types revealed the effects of SAS (1, 16, 18–22). However, it turned out that the cell culture and incubation conditions strongly influence the outcome of *in vitro* tests. While the effect of SiO_2_ is mostly low in the presence of serum proteins, serum-free testing conditions augmented the bioactivity of SAS on cells *in vitro* several-fold (23–28).

Considering silanol groups as reactive sites, several *in vitro* and *in vivo* studies provided evidence that blocking and/or inactivating these reactive groups lowered the bioactivity of crystalline silica such as quartz. As early as 1961, Schlipköter and Brockhaus proposed polyvinylpyridine-N-oxide (PVNO) as a substance mitigating the inflammatory and profibrotic effects of quartz in the lung (29), an effect which also reduced the toxicity of crystalline silica *in vitro* (30, 31). Also, other substances such as Lewis acids were successful in this respect. Comparing the density and steric properties of silanol groups of crystalline and amorphous silica revealed that the hemolytic properties of quartz increase with the number of geminal, but not single silanol groups (32). Only recently, however, a oligomeric, amino-modified siloxane was found to inhibit the *in vitro* and *in vivo* effects of quartz (33).

The effects of organosilane-treated SAS have mainly been studied *in vivo*. Older studies on the acute, subacute, and chronic inhalation exposure of rats mostly to “reaction products of dichlorodimethyl silane (DDS) with silica” are summarized by Becker et al. (34) (see Table 4 of that reference). Overall, these studies showed minor or absent effects in the lower concentration range. Even in cynomolgus monkeys, a concentration of 10 mg/m^3^ administered for 1 year elicited no adverse effects (see Ref. 68 in Becker et al. 2013). Similarly, Lewinson et al. (1994) found no toxicity in rats of DDS-treated hydrophobic amorphous silica [DDS reacts with silanol groups and converts them to methylsilyl groups (-Si-CH_3_)] (35). The concentrations of >30 mg/m^3^ in a 90-day study of organosilane-treated pyrogenic SAS were reported to evoke diffuse or reversible fibrogenesis-like symptoms, but in these cases, the effects were most likely related to high-concentration phenomena (17, 34).

Unlike inhalation experiments, *in vitro* studies offer the possibility to study larger numbers of surface-treated SAS, compare effects of different coating reagents and/or particle sizes, and investigate the cellular modes of action of organosilane-treated SAS. To this end, we studied the cytotoxicity and pro-inflammatory effects of nine surface-treated vs. five untreated SAS with alveolar macrophages. These cells are responsible for clearing inhaled particles from the lung parenchyma where they form a first line of defense against inhaled microorganisms and respirable dusts. Here, we use a well-established *in vitro* test based on the rat alveolar macrophage cell line NR8383 to determine the *in vitro* toxicity of SAS (36). This assay has been validated against 18 short-term inhalation studies and was carried out under protein-free conditions, as this allows to analyze the effects of surface modification without the formation of a protein corona (36). Cytotoxic, activating, and pro-inflammatory effects as well as oxidative stress are analyzed from the cell culture supernatant of cells which are exposed to particles under submersed conditions. However, the dispersion of highly hydrophobic powder materials requires a special strategy. While powders of non-treated (rather hydrophilic) SAS, which contain aggregates and/or agglomerates, can be dispersed with moderate ultrasonic dispersion (USD) energy (3, 37), highly hydrophobic surface-treated SAS demands a different protocol in which particles are pre-wetted with ethanol before they can be immersed in aqueous media, where they are then subjected to extensive USD in the presence of a minimal amount of bovine serum albumin.

Using these dispersion strategies, the particle size distribution and effects of organosilane-treated SAS, as shown in Figure 1, on NR8383 alveolar macrophages will be described and compared to those of more hydrophilic SAS dispersed with both protocols. The results show that hydrophobic surface treatment with organosilanes can abrogate or at least largely diminish the bioactivity of SAS under *in vitro* conditions.

## Materials and Methods

### Particle Properties

Powder materials (12/14) and colloidal SAS delivered as suspension (LUDOX® SM, LUDOX® TM 50, all industrial grades) were provided by members of the consortium SASforREACH GbR as listed in Table 1, together with the details of surface treatment, chemical modification, primary particle, and aggregate sizes, as well as specific surface area according to the Brunauer–Emmett–Teller (BET) method. Micron-sized corundum and quartz DQ12 particles were included in the study as negative and positive particle controls, respectively, as previously described (36, 38).

### Preparation of Particle Suspensions

#### Dispersion of Hydrophobic SAS: Protocol 1

Highly hydrophobic SAS was dispersed according to the NanoGenoTox Protocol (39) for which a minor adaptation was necessary. To achieve a complete wetting of the powders, 15.36 mg of the powder materials was wetted with 60 μl of ethanol (instead of 30 μl). The samples were then mixed with 6 ml of H_2_O containing 0.05% of bovine serum albumin fraction V (BSA), vortexed, and subjected to ultrasonic treatment for 16 min on ice, using a Branson 450D Sonifier, equipped with a 1 cm sonotrode (applied energy density 3,140 J/ml). By this, stable stock suspensions of surface-treated SAS were created (2.56 mg/ml) which were used for all *in vitro* tests. Of note, the final concentrations of ethanol and BSA in all cell assays amounted to 0.04 and 0.002%, respectively, due to further dilution.

#### Dispersion of Hydrophilic SAS: Protocol 2

To prepare particle stock suspensions for cell culture studies from powder materials, 25.6 mg was transferred into 10-ml sterile pyrogen-free H_2_0 (B. Braun Melsungen AG, Melsungen, Germany), vortexed, and ultrasonicated on ice for 15 × 12 s, using a Branson 450D Sonifier, equipped with a 5 mm sonotrode; total ultrasonic energy delivered this way amounted to 270 J/ml (37). For colloidal SAS (LUDOX® SM, LUDOX® TM 50), the concentration was adjusted to 2.56 mg/ml, secondary to a gravimetrical measurement of the dry mass of the original suspensions using a Mettler Toledo AT20 microbalance. All suspensions showed no or only minimal settled material and were used throughout the study.

In an additional set of experiments, hydrophilic SAS was dispersed as described for the hydrophobic SAS and with the same final amounts of BSA and ethanol.

### Measurements of Particle Size Distribution by Particle Tracking Analysis

In addition to the particle sizes provided in Table 1, we determined the particle size distribution in the cell culture medium, that is, under assay conditions by particle tracking analyses (PTA). A NanoSight LM10 instrument equipped with a violet laser (405 nm), an Andor CCD camera, and particle tracking software (NTA 3.0, Malvern Instruments GmbH, Herrenberg, Germany) was used. To measure particle and/or aggregate/agglomerate sizes of SAS suspensions under cell culture conditions, the aqueous particle suspensions were incubated under cell culture conditions (37°C, 5% CO_2_) for 90 min in KRPG, or for 16 h in F-12K medium, respectively. The suspensions were serially diluted with the respective medium (H_2_O, KRPG, or F-12K medium) to optimize the apparent particle concentration to PTA requirements (~5 × 10^8^ particles/ml). The results and respective dilution factors are presented in Table 2. Since the technique is limited by the light-scattering properties of particles, (colloidal) SAS particles smaller than 50 nm were not detected (Supplementary Figures S1, S2).

**Table 2:** Hydrodynamic diameter of SAS particles in H_2_O, KRPG buffer, and F-12K medium.

**Particle Name**	**Protocol**	**Fluid**	**Hydrodynamic diameter [nm]**				
			**Mean ±SEM**	**Mode ±SEM**	**d10 ±SEM**	**d50 ±SEM**	**d90 ±SEM**
CAB-O-SIL® S17D	2	H_2_O	136.3 ± 2.2	125.6 ± 5.8	87.1 ± 0.6	123.8 ± 3.3	182 ± 3.9
	1	H_2_O	136 ± 1.5	117 ± 1	86 ± 0.4	122 ± 1	183 ± 2.4
	2	KRPG	152.3 ± 1.6	135.1 ± 9.6	93.3 ± 1.2	141 ± 1.7	213 ± 9.6
	2	F-12K	224.8 ± 21.5	163.8 ± 22.8	111.7 ± 33.9	213.4 ± 18.5	345.4 ± 24.9
	1	F-12K	149 ± 2.5	134 ± 1	107 ± 2.7	137 ± 3.6	192 ± 3.9
CAB-O-SIL® (E)L-90	2	H_2_O	243.4 ± 1	229.5 ± 17.4	154.8 ± 0.9	226.7 ± 4.6	334.7 ± 2
	1	H_2_O	202 ± 1	169 ± 8.1	132 ± 1.2	188 ± 1.5	284 ± 1.7
	2	KRPG	274.8 ± 14.9	194.7 ± 20.4	172.1 ± 10.7	253.5 ± 16.8	389.7 ± 20.4
	2	F-12K	301.3 ± 15.4	254.6 ± 41.4	182.9 ± 9	289.1 ± 15.5	417.4 ± 21.5
	1	F-12K	260 ± 13	212 ± 35.9	164 ± 8.3	250 ± 11.3	356 ± 17.3
AEROSIL® 50	2	H_2_O	233.7 ± 4	195.3 ± 12.1	148.9 ± 2.9	210.6 ± 2.3	334.2 ± 8.9
	1	H_2_O	221 ± 0.5	183 ± 9.4	139 ± 3.3	205 ± 1.6	305 ± 4.6
	2	KRPG	308.8 ± 6.1	266.9 ± 44.9	195.1 ± 3.8	296.2 ± 16.5	438.2 ± 19
	2	F-12K	297.8 ± 5.2	278.2 ± 6	185.3 ± 5.1	283.6 ± 4.1	409.6 ± 2.7
	1	F-12K	295 ± 9.7	282 ± 11.9	183 ± 6	279 ± 5.2	413 ± 22.5
AEROSIL® R 816	2	H_2_O	206.9 ± 7.1	166.2 ± 12.6	133.4 ± 2.3	191.9 ± 5.8	281.6 ± 13.4
	1	H_2_O	149 ± 2.1	125 ± 5	98 ± 1.4	134 ± 1.4	204 ± 6.3
	2	KRPG	388.9 ± 24.3	297.5 ± 40.9	227.2 ± 15.1	369.1 ± 16.8	559.4 ± 34.2
	2	F-12K	372.9 ± 9.9	334.1 ± 77.1	190.6 ± 6.8	372 ± 10.1	528.9 ± 9
	1	F-12K	241 ± 2.9	152 ± 5.4	134 ± 3.1	202 ± 1.2	396 ± 10.9
CAB-O-SIL® TGC413TRD	1	H_2_O	103.4 ± 0.5	94.3 ± 1.7	68.4 ± 1.5	93.7 ± 1.2	133 ± 1.9
	1	KRPG	88.9 ± 0.4	75.9 ± 2	58.5 ± 1	78.7 ± 0.7	116.3 ± 0.7
	1	F-12K	114.7 ± 1.5	101.9 ± 0.3	67.7 ± 1.5	102 ± 1.2	156.3 ± 2.7
CAB-O-SIL® TS610	1	H_2_O	368.9 ± 1.6	394.1 ± 13.2	211.1 ± 5.1	369.5 ± 2.4	500.7 ± 18.7
	1	KRPG	170.7 ± 0.9	149.5 ± 4.8	112.9 ± 2	156.2 ± 0.9	226.4 ± 2.2
	1	F-12K	182.9 ± 2.7	130.6 ± 6.1	115.9 ± 2.4	166.6 ± 3.2	250.5 ± 3.8
CAB-O-SIL® TS720	1	H_2_O	380.6 ± 12.2	349.3 ± 48.1	233.7 ± 13.9	383.8 ± 11.5	501.5 ± 12.8
	1	KRPG	174.9 ± 1.9	142.8 ± 15.5	115.2 ± 0.2	160.7 ± 3.2	238.2 ± 3.9
	1	F-12K	185.7 ± 1.6	145.3 ± 12	111.6 ± 0.8	162.3 ± 2.2	260 ± 5.7
HDK® H15	1	H_2_O	224.5 ± 5.7	187.6 ± 24.1	136 ± 3	203.3 ± 4.4	334.8 ± 16.2
	1	KRPG	184.1 ± 1.8	141.2 ± 3.2	125.8 ± 1.2	167.8 ± 2.4	249.6 ± 2.3
	1	F-12K	203 ± 1.7	203.3 ± 9.6	129.5 ± 0.4	191 ± 2.5	284.4 ± 4.6
HDK® H2000	1	H_2_O	252.4 ± 4.5	148.2 ± 13.2	115.6 ± 1.1	226.2 ± 3.4	419.3 ± 9.2
	1	KRPG	323.5 ± 13.7	249.3 ± 62.9	147.9 ± 10.3	301.7 ± 17.1	523.4 ± 9.7
	1	F-12K	441.3 ± 9.6	392.2 ± 61.9	204.7 ± 11.5	425.4 ± 6.8	682.4 ± 38.2
AEROSIL® R 504	1	H_2_O	262.9 ± 21.7	170.8 ± 33.8	131.8 ± 15.2	238.7 ± 19.7	407.3 ± 25.2
	1	KRPG	249.2 ± 31	130.6 ± 26.3	121.1 ± 23.6	215.4 ± 34.6	428.2 ± 47.3
	1	F-12K	300.1 ± 2.7	258.4 ± 29	145.4 ± 3.2	279.9 ± 9.7	474.5 ± 16.8
AEROSIL® R 711	1	H_2_O	377.7 ± 10.1	379.7 ± 41.4	216.7 ± 10	374.1 ± 15.5	532.6 ± 12.4
	1	KRPG	179.7 ± 0.8	131.6 ± 5.2	110.1 ± 1	159.6 ± 0.9	263.5 ± 3.6
	1	F-12K	181.5 ± 3.1	137.8 ± 7.3	116.1 ± 0.7	163.7 ± 3.1	262.8 ± 15.5
SIPERNAT® D 17	1	H_2_O	266.8 ± 4.6	183.3 ± 12.7	154.8 ± 1	241.2 ± 2.6	408.7 ± 12.8
	1	KRPG	214.3 ± 1.4	154.5 ± 6.1	136 ± 1.2	186.9 ± 2.5	319.4 ± 9.9
	1	F-12K	198.8 ± 4	160.2 ± 8.3	122.7 ± 1	172 ± 2.7	295.6 ± 16.2

*Particles were dispersed in H_2_O following Protocol 1 or 2 as indicated, diluted in either H_2_O, KRPG, or F-12K medium to 90 μg/ml and incubated at 37°C for 90 ± 20 min (H_2_O, KRPG) or 16 ± 1 h (F-12K medium) to mimic culture conditions. HDs are means ± SD from three technical replicates. No background correction was carried out because H_2_O, KRPG, or serum-free F-12K medium contained no PTA-detectable particles. Values for d10, d50, and d90 describe the cumulative particle size distribution at 10, 50, and 90% of the maximum value*.

### Sterility Testing

To test for any fungal or bacterial contaminations, 100 μl of the final aqueous suspension as prepared for *in vitro* testing was plated onto caso agar and malt extract agar (both from Applichem GmbH, Darmstadt, Germany) and incubated at 37°C for 3 days. Neither bacterial nor fungal contaminants were detected.

### Cell Culture and *in vitro* Testing

NR8383 cells (ATCC, USA; ATCC® Number: CRL-2192TM) were cultivated in F-12K medium supplemented with 15% fetal calf serum (FCS), 100 μg/ml streptomycin, 100 U/ml penicillin, and 2 mM L-glutamine (all from PAN Biotech, Aidenbach, Germany) as described.

Assays were carried out as described (36). Cells were seeded into 96-well plates (3 × 10^5^ cells/well) and kept at 37°C and 5% CO_2_. Each well contained 200 μl F-12K cell culture medium in which the concentration of FCS was reduced to 5%. After 24 h, the medium was replaced by serum-free suspensions of the test materials, which were diluted to 90, 45, 22.5, and 11.25 μg/ml either with KRPG buffer (129 mM NaCl, 4.86 mM KCl, 1.22 mM CaCl_2_, 15.8 mM NaH_2_PO_4_, 5–10 mM glucose; pH 7.3–7.4) or with serum-free F-12K medium.

To measure the release of H_2_O_2_, the particles were administered in KRPG buffer. Released H_2_O_2_ was measured after 90 min using the Amplex Red® assay. The optical density of resorufin was measured photometrically at 570 nm (reference value: 620 nm) with a plate reader (Tecan Infinite F200Pro, Tecan GmbH, Germany). Positive controls were run with zymosan (180 μg/ml). All values were corrected for background absorbance using cell-free particle controls and converted into absolute concentrations of H_2_O_2_ using the molar extinction coefficient of resorufin (54,000 L × mol^−1^ × cm^−1^).

To determine the release of LDH, GLU, and TNFα from the cells, the test materials were administered in serum-free F-12K medium and supernatants were retrieved after 16 h. LDH activity was measured using the Roche Cytotoxicity Kit (Sigma-Aldrich, Taufkirchen, Germany). To measure GLU activity, the supernatant (50 μl) was mixed with 100 μl 0.2 M sodium acetate buffer (pH 5) containing 13.3 mM p-nitrophenyl-D-glucuronide and 0.1% Triton X-100. The color reaction was stopped with 100 μl 0.2 M NaOH terminated; the optical density was measured at 405 nm. LDH and GLU measurements were background corrected and normalized to the positive control values (set to 100%) obtained by lysing the cells with 0.1% Triton X-100 in F-12K.

The concentration of tumor necrosis factor α (TNFα) was determined with a specific enzyme-linked immunosorbent assay (ELISA) for rat TNFα (Quantikine ELISA Kit, Bio-Techne GmbH, Wiesbaden-Nordenstadt, Germany). The TNFα-forming capacity of NR8383 cells was controlled by adding lipopolysaccharide (0.1 μg/ml, Sigma-Aldrich, Taufkirchen, Germany).

In all assays, cell controls were carried out by adding particle-free vehicle, that is, F-12K medium only, or F12-K medium containing BSA and ethanol in concentrations as stated above.

### Light Microscopy

To supplement the size data from PTA analysis, to verify particle uptake by cells, and to describe cell morphology, phase contrast micrographs were taken using a Zeiss Axiovert 40-C Microscope. Gravitationally settled particles were micro-graphed under cell culture conditions in the absence of cells with a Nikon BioStation equipped with a 20 × phase contrast optics.

### Statistical Evaluation

*in vitro* data were generated in triplicates, and three independent repetitions were carried out. To test for significant differences, values from each concentration were compared to non-treated controls using two-way analysis of variance (ANOVA) with Dunnett's multiple comparison test. A value of *P* ≤ 0.05 was considered significant (^*^). All data were expressed as mean ± standard deviation (SD). All calculations were carried out with GraphPad Prism software.

## Results

### Size Determination of SAS in Cell Culture Experiments

*in vitro* effects of particles on phagocytic cells depend on particle size and gravitational settling. To obtain insight into the so-called particokinetics (40) of surface-treated and unmodified SAS, we measured the hydrodynamic diameter (HD) of suspended particles under cell culture conditions. Particle tracking analysis (PTA) was used as it allows to detect light-scattering nanoparticles at relatively low concentration.

The HD data, from size distribution plots (Supplementary Figures S1, S2), are summarized in Table 2, where the (ultra)fine fraction of SAS particles primarily dispersed in H_2_O is compared to the HD found in KRPG buffer after 90 min and in F-12K medium after 16 h, respectively. The non-treated SAS CAB-O-SIL® S17D Fumed Silica (CAB-O-SIL® S17D), CAB-O-SIL® (E)L-90, and AEROSIL® 50, as well as the slightly hydrophilic surface-treated AEROSIL® R 816, when dispersed with Protocol 2, tended to slightly agglomerate. This was reflected by an increase in HD (mode value) of 42.4% for AEROSIL® 50 and 101.0% for AEROSIL® R 816 (Table 2 and Supplementary Table S1). Hydrophobic surface-treated *SAS*, which had to be dispersed with Protocol 1, behaved less uniform, since we observed increases in HD of up to 164.6% (HDK® H2000) and decreases down to −63.7% (AEROSIL® R 711).

**Figure 2:**
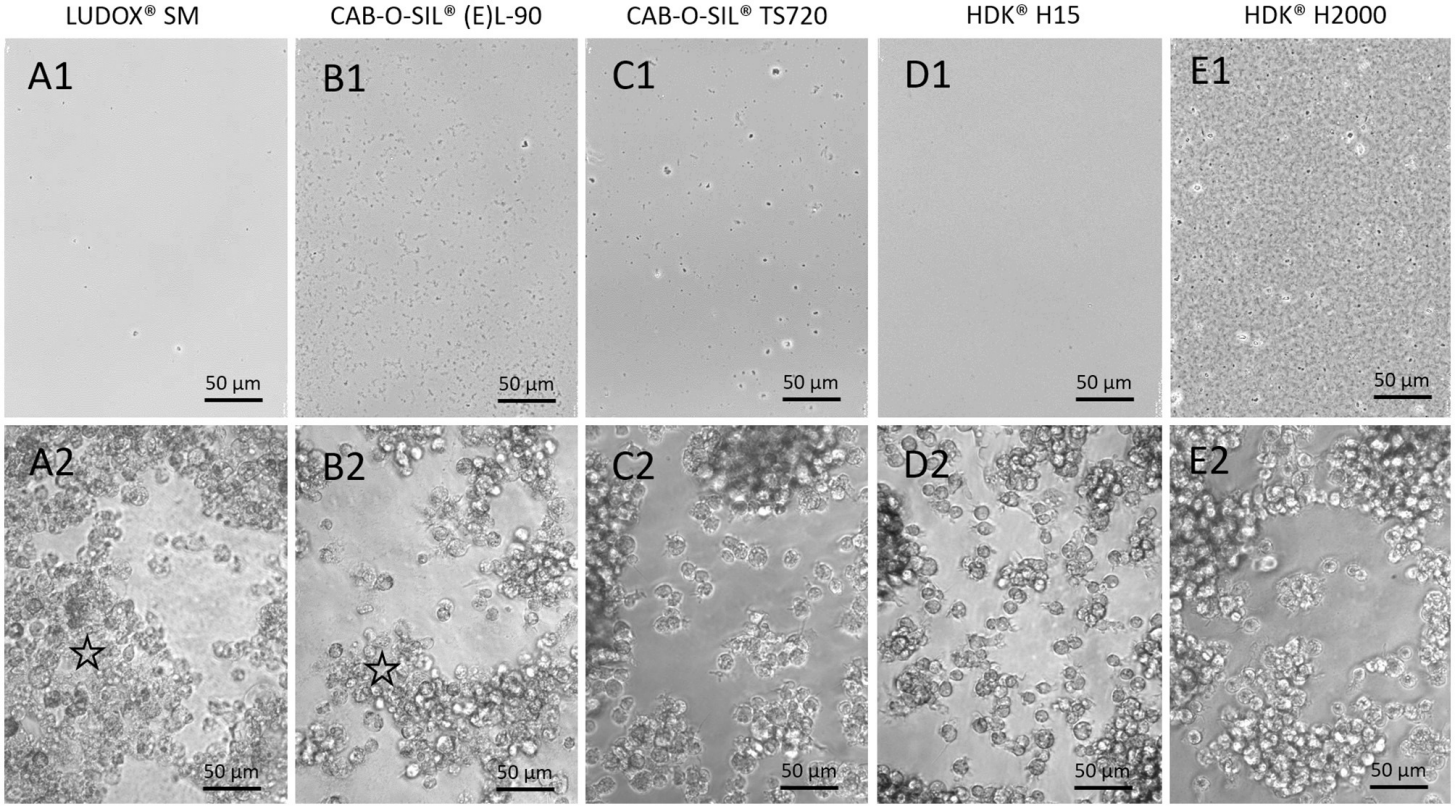
Gravitational settling and uptake of selected SAS. Phase contrast images from cell culture experiments with 90 μg/ml of indicated SAS in the absence (A1–E1, cell-free controls) and presence of NR8383 cells (A2–E2). **(A,B)** Untreated SAS dispersed with Protocol 2, **(C–E)** surface-treated SAS dispersed with Protocol 1. Note that particle settling is obvious at different degrees in cell-free controls (A1–E1). In the presence of cells, precipitates are no longer visible indicating particle uptake. Areas with granular, deteriorated cells (asterisks) are seen in the presence of untreated SAS only. See text for further explanation.

In line with these measurements, most SAS did not further agglomerate under cell culture conditions. Nevertheless, phase contrast microscopy revealed low numbers of aggregates/agglomerates (e.g., CAB-O-SIL® TS 720, CAB-O-SIL™ TGC413TRD treated silica) or a thin layer of loose precipitates (CAB-O-SIL® (E)L-90, AEROSIL® R 816, HDK® 2000, and AEROSIL® R 504) all of which were visible under cell-free conditions after 16 h of incubation (Figure 2 and Supplementary Figures S4, S5). Overall, gravitational settling of SAS tested in this study was very limited.

### *In vitro* Effects of SAS on Alveolar Macrophages

To study the biological activity of untreated and surface-treated SAS *in vitro*, we used the well-established alveolar macrophage test, which analyzes the releases of lactate dehydrogenase (LDH), glucuronidase (GLU), tumor necrosis factor α (TNFα), and H_2_O_2_ from NR8383 cells into the cell culture supernatants. Vehicle-treated cells were used as negative controls. Micron-sized corundum and quartz DQ12 (0–180 μg/ml) were used as negative and positive benchmark controls.

To compare *in vitro* effects of surface-treated and untreated SAS, we administered serially diluted particle suspensions (11.25–90 μg/ml) to the cells. Particle uptake was confirmed by light microscopy at least for the gravitationally settled aggregates/agglomerates which are visible with phase contrast optics (Figures 2B,E and Supplementary Figures S3–S5).

#### Controls

While corundum elicited no adverse responses in all tests, quartz DQ12 led to cytotoxicity, activation, and pro-inflammatory response indicated by increased release of LDH, GLU, and TNFα, respectively (Table 3). The TNFα responsiveness of the cells was controlled with lipopolysaccharide (LPS, 0.5 μg/ml) which increased TNFα concentration to 1,027 ± 164 pg/ml. The induced formation of H_2_O_2_ was typically low upon both corundum and quartz DQ12 (<1.5 μM), but increased to 23.4 ± 2.0 μM upon zymosan, due to its well-known induction of the NADPH oxidase reaction in macrophages. Overall, the effects of cell and particle controls were within the borders of our historical records, indicating the responsiveness of NR8383 macrophages and the validity of the assay.

**Table 3:** Effects of non-treated SAS dispersed with Protocol 1 and 2 on NR8383 cells.

**Concentration**	**[μg/ml]**	**Protocol**	**LDH**	**GLU**	**H_**2**_O_**2**_**	**TNFα**
			**[% pos. Control]**	**% pos. Control**	**[μmol/L]**	**[pg/ml]**
			**Mean ±SD**	**Mean ±SD**	**Mean ±SD**	**Mean ±SD**
Corundum	0	2	10.3 ± 2.4	1.6 ± 0.2	0.8 ± 0.1	37.6 ± 23.6
	22.5	2	7.8 ± 1.4	1.3 ± 0.1	0.9 ± 0.1	28.5 ± 11.2
	45	2	9.3 ± 1.2	2.2 ± 1.6	1.0 ± 0.0	32.5 ± 13.6
	90	2	11.1 ± 1.1	2.6 ± 2.2	1.0 ± 0.1	33.5 ± 15.8
	180	2	12.8 ± 1.3	2.5 ± 1.8	1.2 ± 0.3	36.8 ± 21.4
Quartz DQ12	0	2	10.3 ± 2.4	1.6 ± 0.2	0.8 ± 0.1	37.6 ± 23.6
	22.5	2	9.1 ± 0.9	2.4 ± 1.6	1.0 ± 0.2	31.9 ± 17.2
	45	2	11.7 ± 1.9	1.2 ± 0.6	1.0 ± 0.2	45.4 ± 23.3
	90	2	27.2 ± 4.0***	3.6 ± 0.7	1.1 ± 0.2	90.7 ± 41.4
	180	2	64.2 ± 3.4***	12.5 ± 1.2***	1.3 ± 0.7	233.0 ± 93.1***
LUDOX® SM	0	1	15.1 ± 4.3	1.6 ± 0.4	–	–
	11.25	1	31.9 ± 11.4*	2.8 ± 0.6	–	–
	22.5	1	46.0 ± 13.0***	4.6 ± 1.0	–	–
	45	1	79.0 ± 22.0***	9.5 ± 2.4	–	–
	90	1	98.8 ± 17.3***	15.2 ± 1.2	–	–
	0	2	10.3 ± 2.4	1.6 ± 0.2	0.8 ± 0.1	37.6 ± 23.6
	11.25	2	14.4 ± 4.2	2.0 ± 0.4	0.8 ± 0.1	47.2 ± 14.3
	22.5	2	40.2 ± 7.7***	2.7 ± 4.1	0.8 ± 0.2	165.5 ± 47.8*
	45	2	77.6 ± 9.2***	11.9 ± 2.4***	0.8 ± 0.4	479.1 ± 250.8***
	90	2	89.3 ± 5.1***	14.1 ± 2.1***	0.8 ± 0.7	541.4 ± 111.1***
LUDOX® TM-50	0	1	15.1 ± 4.3	1.6 ± 0.4	–	–
	11.25	1	15.6 ± 3.8	1.4 ± 0.3	–	–
	22.5	1	19.9 ± 7.5	1.7 ± 0.7	–	–
	45	1	50.5 ± 13.3***	4.2 ± 0.9**	–	–
	90	1	86.3 ± 6.6***	9.5 ± 1.7***	–	–
	0	2	10.3 ± 2.4	1.6 ± 0.2	0.8 ± 0.1	37.6 ± 23.6
	11.25	2	14.8 ± 6.2	1.6 ± 0.7	0.8 ± 0.1	110.8 ± 26.0
	22.5	2	42.8 ± 7.9***	4.2 ± 0.9*	0.9 ± 0.3	299.4 ± 4.5***
	45	2	75.7 ± 8.8***	12.1 ± 1.9***	1.1 ± 0.7	449.5 ± 99.2***
	90	2	84.8 ± 12.7***	17.4 ± 1.9***	1.0 ± 0.8	202.8 ± 120.2**
CAB-O-SIL® S17D	0	1	15.1 ± 4.3	1.6 ± 0.4	–	–
	11.25	1	15.6 ± 5.4	1.7 ± 0.5	–	–
	22.5	1	53.0 ± 15.2***	6.6 ± 1.5***	–	–
	45	1	92.7 ± 14.2***	15.2 ± 1.3***	–	–
	90	1	95.5 ± 15.8***	15.5 ± 1.2***	–	–
	0	2	9.5 ± 1.8	1.3 ± 0.4	0.8 ± 0.2	49.3 ± 19.5
	11.25	2	82.8 ± 3.3***	17.4 ± 1.0***	1.5 ± 0.2	537.2 ± 210.2***
	22.5	2	81.3 ± 7.3***	15.0 ± 0.2***	2.2 ± 0.5***	412.9 ± 15.7***
	45	2	63.9 ± 8.0***	11.8 ± 0.4***	2.8 ± 0.9***	242.8 ± 7.5***
	90	2	33.2 ± 3.3***	7.2 ± 0.2***	2.8 ± 1.3***	92.5 ± 25.4
CAB-O-SIL® (E)L-90	0	1	15.1 ± 4.3	1.6 ± 0.4	–	–
	11.25	1	14.1 ± 4.2	1.5 ± 0.3	–	–
	22.5	1	18.5 ± 5.6	2.0 ± 0.7	–	–
	45	1	81.9 ± 22.8***	12.6 ± 3.6***	–	–
	90	1	101.7 ± 15.8***	23.6 ± 0.8***	–	–
	0	2	9.5 ± 1.8	1.3 ± 0.4	0.8 ± 0.2	49.3 ± 19.5
	11.25	2	24.6 ± 10.7***	3.9 ± 1.7*	1.0 ± 0.1	128.2 ± 38.6
	22.5	2	75.7 ± 4.7***	15.3 ± 1.8***	0.9 ± 0.4	252.5 ± 22.4***
	45	2	80.5 ± 5.1***	18.6 ± 0.9***	1.0 ± 0.7	216.7 ± 19.4***
	90	2	80.6 ± 6.3***	18.1 ± 0.4***	1.0 ± 0.9	175.5 ± 23.3*
AEROSIL® 50	0	1	13.2 ± 4.6	1.4 ± 0.2	–	–
	11.25	1	12.0 ± 4.1	0.9 ± 0.5	–	–
	22.5	1	12.5 ± 3.7	1.9 ± 0.1	–	–
	45	1	23.2 ± 8.2	1.9 ± 0.5	–	–
	90	1	90.0 ± 9.7***	3.5 ± 1.4	–	–
	0	2	9.5 ± 1.8	1.3 ± 0.4	0.8 ± 0.2	49.3 ± 19.5
	11.25	2	11.1 ± 4.1	1.8 ± 0.4	0.9 ± 0.2	58.9 ± 17.0
	22.5	2	41.8 ± 13.4***	7.0 ± 2.4***	1.0 ± 0.3	188.0 ± 32.1**
	45	2	87.4 ± 5.7***	18.3 ± 1.9***	1.3 ± 0.8	289.8 ± 42.9***
	90	2	87.5 ± 0.6***	19.0 ± 1.6***	1.4 ± 1.1	226.9 ± 40.7***

Mean values and standard deviations from three independent experiments. LDH, lactate dehydrogenase; GLU, glucuronidase; ROS, reactive oxygen species (H_2_O_2_); TNF, tumor necrosis factor α (TNFα). Values significantly different from cell control are marked by asterisks (*P ≤ 0.05, **P ≤ 0.01, ***P ≤ 0.001). Two-way analyses of variance (ANOVA) and Dunnett's test were used to compare means from the control and treated groups. n.m., values not measured.

#### Untreated SAS

In general, the majority of untreated SAS, when dispersed with Protocol 2 (Table 3), induced a largely similar pattern of responses characterized by dose-dependent cytotoxic effects (LDH) and releases of GLU. Low-observed effect concentrations (LOECs) were generally ≤ 22.5 μg/ml for LDH and 22.5–45 μg/ml for GLU. Unexpectedly, CAB-O-SIL® S17D elicited an inverse dose response in both assays, starting with a full-blown response upon 11.25 μg/ml. The induction of TNFα was mostly biphasic with a maximum between 22.5 and 45 μg/ml, and LOECs ranged from 11.25 (CAB-O-SIL® S17D) to 22.5 μg/ml (other untreated materials). A significant dose-dependent release of H_2_O_2_ was found for CAB-O-SIL® S17D at concentrations ≥22.5 μg/ml. All other untreated or hydrophilic SAS elicited no change in the extracellular H_2_O_2_ concentration.

Untreated SAS was also dispersed according to Protocol 1 to compare the releases of LDH and GLU to those induced by surface-treated SAS. The effects of CAB-O-SIL® S17D, CAB-O-SIL® (E)L-90, AEROSIL® 50, and LUDOX® TM 50 were strongly mitigated, while the effects of both LUDOX® variants were less affected or remained even identical (Table 3). Of note, ethanol wetting together with pronounced ultrasonic treatment in the presence of low amount of BSA (Protocol 1) shifted the dose–response curves rightwards (Supplementary Figures S6, S7), thus increasing EC_50_ values up to 2.8-fold with colloidal SAS being least affected (Table 4). Importantly, the application of Protocol 1 to hydrophilic SAS did not reduce the bioactivity down to control level as observed for highly hydrophobic SAS. Overall, untreated SAS exhibited a high bioactivity in the alveolar macrophage assay.

**Table 4:** EC_50_ values of LDH and GLU release obtained with Protocol 1 or 2.

		**EC50 LDH release [μg/ml]**			**EC50 GLU release [μg/ml]**		
		**Protocol 1**	**Protocol 2**	**Fold change**	**Protocol 1**	**Protocol 2**	**Fold change**
LUDOX® SM	EC_50_	26.3	26.1	1.0	37.5	34.2	1.1
	R^2^	0.9	1.0		0.9	0.9	
LUDOX® TM-50	EC_50_	44.3	24.4	1.8	49.9	36.4	1.4
	R^2^	0.9	1.0		0.9	1.0	
CAB-O-SIL® S17D*	EC_50_	23.2	–	–	25.0	–	–
	R^2^	0.9	–		1.0	–	
CAB-O-SIL® (E)L-90	EC_50_	37.1	14.2	2.6	44.7	15.9	2.8
	R^2^	0.9	1.0		1.0	1.0	
AEROSIL® 50	EC_50_	58.0	24.1	2.4	53.6	26.3	2.0
	R^2^	1.0	1.0		0.6	1.0	

*No curve could be fitted to the data from Protocol 2. All EC_50_ values were calculated for a 95% confidence interval.

#### Surface-Treated SAS

**Figure 3:**
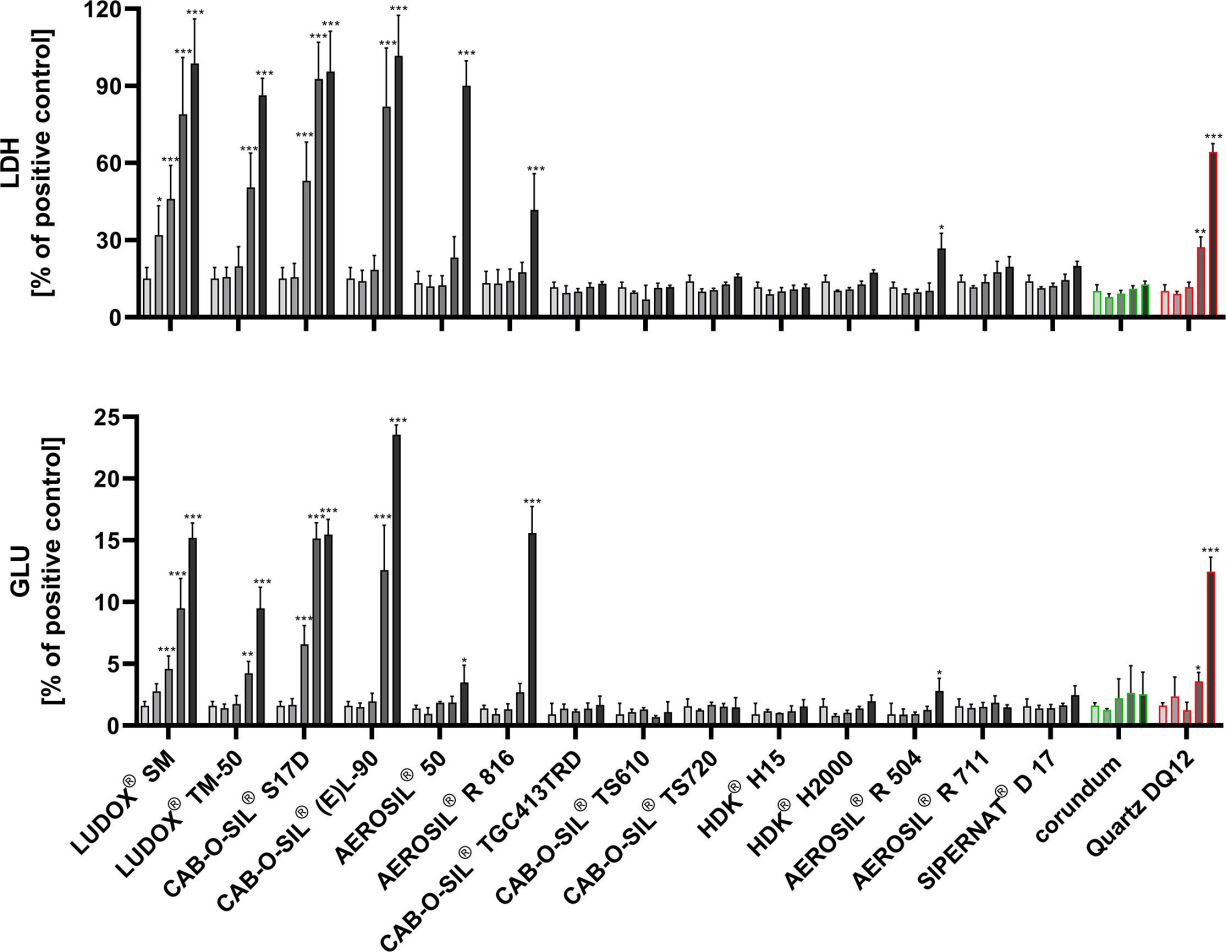
Comparison of the *in vitro* response of NR8383 alveolar macrophages to untreated and surface-treated SAS. All SASs were dispersed with Protocol 1 (columns from left to right: 0, 11.25, 22.5, 45, and 90 μg/ml). (Upper panel) lactate dehydrogenase activity (LDH); (lower panel) glucuronidase activity (GLU); LDH and GLU values are shown relative to the triton X-100 positive control. Untreated SAS: LUDOX® SM, LUDOX® TM 50, CAB-O-SIL® S17D, CAB-O-SIL® (E)L-90, AEROSIL® 50. Surface-treated SAS: AEROSIL® R 816, CAB-O-SIL® TGC413TRD, CAB-O-SIL® TS610, CAB-O-SIL® TS720, HDK® H15, HDK® H2000, AEROSIL® R 504, AEROSIL® R 711, SIPERNAT® D 17. Corundum and quartz DQ12 particles (0, 22.5, 45, 90, and 180 μg/ml) were used as negative and positive controls. Values significantly different from cell control are marked by asterisks (*P ≤ 0.05, **P ≤ 0.01, ***P ≤ 0.001).

The majority of hydrophobic SAS, when dispersed with Protocol 1, elicited no changes in LDH, GLU, and TNFα (Table 5). AEROSIL® R 504, whose surface treatment combines HDMZ with AMEO, induced a small increase in LDH and GLU, which was significant for LDH upon 90 μg/ml only. Another exception was the slightly hydrophilic AEROSIL® R 816, whose effects resembled the non-treated SAS (c.f. Table 3). There were also no results pointing to an induction of H_2_O_2_ formation by surface-treated hydrophobic SAS. However, some hydrophobic variants decreased the small resorufin signal below cell controls (Table 5). This phenomenon was interpreted as an assay interference (possibly caused by adsorption of colored reactants to hydrophobic surfaces). Taken together, surface-treated hydrophobic SAS showed no or a very limited biological activity. The direct comparison of untreated and surface-treated SAS is shown in Figure 3.

**Table 5:** Effects of surface-treated SAS on NR8383 cells.

**Concentration**	**[μg/ml]**	**Protocol**	**LDH**	**GLU**	**H_**2**_O_**2**_**	**TNFα**
			**[% pos. Control]**	**% pos. Control**	**[μmol/L]**	**[pg/ml]**
			**Mean ±SD**	**Mean ±SD**	**Mean ±SD**	**Mean ±SD**
AEROSIL® R 816	0	1,2^#^	13.2 ± 4.6	1.4 ± 0.3	0.8 ± 0.2	49.3 ± 19.5
	11.25	1,2^#^	13.2 ± 5.4	0.9 ± 0.4	1.0 ± 0.2	38.2 ± 12.1
	22.5	1,2^#^	14.2 ± 4.6	1.3 ± 0.4	1.0 ± 0.2	53.0 ± 8.4
	45	1,2^#^	17.6 ± 3.7	2.7 ± 0.7	1.0 ± 0.4	164.7 ± 36.6*
	90	1,2^#^	41.8 ± 14.8***	15.9 ± 2.5***	1.2 ± 0.8	201.2 ± 16.6**
CAB-O-SIL® TGC413TRD	0	1	11.7 ± 2.0	0.9 ± 0.9	0.8 ± 0.1	47.5 ± 38.0
	11.25	1	9.4 ± 2.8	1.4 ± 0.4	0.5 ± 0.0	58.2 ± 54.8
	22.5	1	9.9 ± 1.2	1.2 ± 0.2	0.3 ± 0.1	56.0 ± 50.2
	45	1	11.9 ± 1.5	1.4 ± 0.5	0.0 ± 0.4	61.8 ± 53.3
	90	1	13.1 ± 0.8	1.7 ± 0.7	n.m.	61.3 ± 53.1
CAB-O-SIL® TS610	0	1	11.7 ± 2.0	0.9 ± 0.9	0.8 ± 0.1	47.5 ± 38.0
	11.25	1	9.6 ± 0.5	1.1 ± 0.2	0.5 ± 0.1	60.3 ± 63.3
	22.5	1	7.0 ± 5.4	1.3 ± 0.2	0.4 ± 0.2	56.3 ± 57.4
	45	1	11.4 ± 1.8	0.7 ± 0.1	0.1 ± 0.2	60.7 ± 53.9
	90	1	11.8 ± 0.7	1.1 ± 0.8	n.m.	61.6 ± 56.7
CAB-O-SIL® TS720	0	1	14.0 ± 2.3	1.6 ± 0.6	0.9 ± 0.1	52.2 ± 25.5
	11.25	1	10.0 ± 1.1	1.2 ± 0.1	0.8 ± 0.0	49.2 ± 22.5
	22.5	1	10.5 ± 0.7	1.7 ± 0.2	0.7 ± 0.1	44.3 ± 23.3
	45	1	12.7 ± 0.9	1.5 ± 0.3	0.4 ± 0.3	47.1 ± 25.4
	90	1	15.8 ± 1.0	1.5 ± 0.8	0.2 ± 0.3	47.4 ± 25.7
HDK® H15	0	1	11.7 ± 2.0	0.9 ± 0.9	0.8 ± 0.1	47.5 ± 38.0
	11.25	1	9.0 ± 1.6	1.2 ± 0.2	0.6 ± 0.1	56.6 ± 45.4
	22.5	1	10.0 ± 1.5	1.0 ± 0.0	0.2 ± 0.1	51.3 ± 32.6
	45	1	10.8 ± 1.6	1.2 ± 0.4	n.m.	57.7 ± 47.5
	90	1	11.6 ± 1.3	1.6 ± 0.5	n.m.	58.3 ± 47.8
HDK® H2000	0	1	14.0 ± 2.3	1.6 ± 0.6	0.9 ± 0.1	52.2 ± 25.5
	11.25	1	10.4 ± 0.2	0.8 ± 0.2	0.9 ± 0.0	43.1 ± 27.8
	22.5	1	10.8 ± 0.7	1.0 ± 0.2	0.7 ± 0.2	43.6 ± 28.8
	45	1	12.8 ± 1.2	1.4 ± 0.1	0.7 ± 0.5	44.8 ± 29.6
	90	1	17.4 ± 1.1	2.0 ± 0.5	0.8 ± 0.6	39.5 ± 31.6
AEROSIL® R 504	0	1	11.7 ± 2.0	0.9 ± 0.9	0.8 ± 0.1	47.5 ± 38.0
	11.25	1	9.3 ± 1.6	0.9 ± 0.5	0.6 ± 0.1	51.1 ± 47.7
	22.5	1	9.7 ± 1.1	0.9 ± 0.2	0.3 ± 0.1	58.8 ± 49.1
	45	1	10.4 ± 2.9	1.3 ± 0.3	0.1 ± 0.1	56.8 ± 43.8
	90	1	26.7 ± 6.0***	2.8 ± 1.0	0.2 ± 0.2	65.6 ± 52.5
AEROSIL® R 711	0	1	14.0 ± 2.3	1.6 ± 0.6	0.9 ± 0.1	52.2 ± 25.5
	11.25	1	11.6 ± 0.6	1.4 ± 0.3	0.6 ± 0.1	57.2 ± 23.4
	22.5	1	13.7 ± 2.7	1.5 ± 0.4	0.4 ± 0.2	55.7 ± 26.7
	45	1	17.6 ± 4.2	1.8 ± 0.6	0.1 ± 0.5	58.5 ± 30.1
	90	1	19.7 ± 3.9	1.5 ± 0.2	n.m.	56.3 ± 33.9
SIPERNAT® D 17	0	1	14.0 ± 2.3	1.6 ± 0.6	0.9 ± 0.1	52.2 ± 25.5
	11.25	1	11.4 ± 0.5	1.4 ± 0.2	0.7 ± 0.2	50.2 ± 30.4
	22.5	1	12.2 ± 1.0	1.4 ± 0.3	0.5 ± 0.2	51.4 ± 35.4
	45	1	14.4 ± 2.4	1.6 ± 0.2	0.3 ± 0.4	54.6 ± 39.0
	90	1	20.0 ± 1.8	2.5 ± 0.7	0.1 ± 0.5	53.2 ± 36.1

Mean values and standard deviations from (n = 3) experiments. LDH, lactate dehydrogenase; GLU, glucuronidase; ROS, reactive oxygen species (H_2_O_2_); TNF α, tumor necrosis factor α. Values significantly different from cell control are marked by asterisks (*P ≤ 0.05, **P ≤ 0.01, ***P ≤ 0.001). Slightly negative values (n.v.) resulting from color interference and background subtraction were omitted. ^#^Values for H_2_O_2_ and TNFα were obtained with Protocol 2.

## Discussion

In this study, we showed that surface-treated highly hydrophobic SAS elicits neither cytotoxic nor pro-inflammatory effects in alveolar macrophages *in vitro*. While untreated SAS was highly bioactive under the serum-free testing conditions, thus inducing the release of LDH, GLU, TNFα, and, partially, also of H_2_O_2_ from NR8383 alveolar macrophages, the organosilane-treated SAS elicited no such effects up to 45 μg/ml, and only two materials showed minor effects at the highest dose (90 μg/ml). This is a striking finding because the effects of many poorly soluble nanomaterials on cells or in the lung correlate with the specific BET surface areas (41) which are comparatively large (BET: 58–200 m^2^/g) for surface-treated SAS of this study. With respect to SAS, it should be added, however, that the bioactivity seen in *in vitro* and *in vivo* studies is not merely a function of the BET surface, especially if pyrogenic, precipitated, gel and colloidal SAS are compared (20, 37, 42). Moreover, this is most likely influenced by surface characteristics (e.g., steric properties of silanol groups) as well as the occurrence of micro- and mesopores.

Our previous studies with the same cell culture model have shown that NR8383 cells are highly sensitive to SAS (28, 36, 37). Accordingly, the EC_50_ values for the release of LDH, which indicate membrane damage and cytotoxicity, were in the range of 7–30 μg/ml for most non-treated SAS in the absence of protein (37). Considering that particles inside the lung will come into contact with the lung surfactant and proteins of the lung lining fluid, the omission of protein under testing conditions may be regarded a somewhat artificial situation. However, as an established routine method, it allows to disclose biological effects of different particle surfaces (43).

To use the test system for highly hydrophobic surface-treated SAS, particles had to be wetted with ethanol and subjected to very high USD energy in the presence of a low, stabilizing concentration of BSA. One might object that the missing bioactivity of surface-treated SAS, when compared to untreated SAS, mainly results from a lowered particle size which might have caused lower gravitational settling and, thus, reduced particle uptake. However, HD values in the cell culture medium were similar in both groups (mode values were 163–278.2 and 101.9–392 nm for untreated and surface-treated SAS, respectively) suggesting a very similar particokinetics of both types of SAS. Also the concentration ranges in the cell culture medium of both types of particles were highly similar, as estimated from the PTA measurements (Supplementary Figures S1, S2, and S8). Overall, the PTA data suggest that there were no major differences in size and number of the small (<500 nm) particles. SAS particles can hardly be viewed inside cells by conventional light microscopy, and the measurement of silicon inside cells requires advanced methods (28) and was not carried out in this investigation. Therefore, indirect methods may be used to estimate the SAS particle concentration inside cells: Particle sedimentation models (44) have shown that SAS with a primary particles sizes of 15–50 nm resulted in settled fractions of 15.8–55% under cell culture conditions (42). The direct measurement with high-resolution ICP-MS showed that 20 or 60%, for example, of pyrogenic AEROSIL® 380 or precipitated SIPERNAT® 160 were associated with NR8383 cells (28). While these data show that the uptake of the small SAS particle fraction is most likely incomplete, settled agglomerates of both untreated (e.g., CAB-O-SIL® (E)L-90) and surface-treated highly hydrophobic particles (e.g., HDK® H2000) were found to be completely cleared from the bottom of the cell culture vessels. This observation shows that organosilane coating does not prevent the ingestion of surface-treated SAS by NR8383macrophages *per se* and suggests that an uptake of the diffusible fraction of hydrophobic SAS is highly likely as well. Taken together, the missing effect of surface-treated highly hydrophobic SAS on LDH, GLU, and TNFα is unlikely caused by a lowered particle uptake. Instead, surface properties need to be considered.

Two aspects of the surface treatment of SAS with organosilanes shall be discussed. The first aspect is the covalent chemical modification itself, which certainly lowers the number of accessible silanol groups. These functional groups have been identified as reactive sites especially in crystalline silica (41) where they can be neutralized with polyvinylpyridine-N-oxide (PVPNO) *in vivo* (29, 30, 33). While the capping of silanol groups may convincingly explain the reduction of acute bioactivity of surface-treated SAS, dissolution rate and long-term stability of surface-modified SAS may be involved as well, especially dissolution rates depend not only on the type of coating but also on curvity and other factors (45, 46) of the core material, such that coated material may display an altered dissolution rate in biological fluids (46). Although the solubility of SAS is lowest at physiological pH and also at pH 4.5 which is typically found inside phagolysosomes (45, 46), the persistence and stability of the different covalently bound organic residues (see Table 1) in biological systems is an open question. A former 90 days inhalation study on rats reported that the organosilane-treated AEROSIL® R 974, which is highly similar to the CAB-O-SIL® TS610 used here, persisted longer in the lung than non-treated SAS (47). At the same time, AEROSIL® 200, AEROSIL® R 974 failed to attract neutrophilic granulocytes directly after exposure indicating absence of inflammatory reaction. Because an erroneously reported pro-fibrotic effect of AEROSIL® R 974 was refuted later (17), the appropriate interpretation of the study of Reuzel et al. (47) is that organosilane treatment can in fact dampen the transient pro-inflammatory effects of untreated SAS in the lung. However, it should be kept in mind that the *in vitro* test as carried out here describes the acute situation only and awaits further confirmation as to whether organosilane coating remains stable under physiological conditions.

In an intratracheal instillation study, a 15-nm-sized colloidal silica and its phosphonated variant were compared for inflammatory effects and local phospholipid distribution after intratracheal instillation of 0.36 mg per rat lung (43). Also, this surface treatment abolished the pro-inflammatory effect of the colloidal silica. In the accompanying *in vitro* study, cytotoxic and pro-inflammatory effects on NR8383 cells were clearly diminished, though not fully suppressed. In this context, it is important to note that phosphonated silica particles were hydrophilic, suggesting that hydrophobicity adds to the diminution of SAS effects.

The cellular mode of actions of SAS has been extensively studied by Karkossa et al. (48). Using two Pluronic F108 dispersed hydrophobic AEROSIL variants coated with organosilane (TMS2 and TMS3), they showed that the hydrophobic surface treatment abolished any effect on both the proteome or metabolome of RLE-6TN cells at a concentration of 10 μg/cm^2^. This result is perfectly in line with our findings and underlines the biocompatibility of organosilane-treated SAS.

The second aspect of the hydrophobic surface treatment concerns the small protein concentration used to disperse hydrophobic particles in cell culture media. It is known that serum proteins, or more precisely the protein corona formed thereof, lower the bioactivity of SAS *in vitro* (24, 25, 27). However, we found that this effect is not uniform for all SAS (28). While the addition of a serum concentration typically used in cell cultures (10%, v/v) clearly inhibited the bioactivity of a SIPERNAT® 50 (a precipitated SAS) without influencing uptake and subcellular distribution of particles, the latter aspects were different for AEROSIL® 50 (a pyrogenic SAS), thus complicating the interpretation of *in vitro* findings. Of note, low concentrations of BSA (<1% w/v) hardly reduced the bioactivity of hydrophilic SAS (data not shown) such that the effects of 0.05% BSA (Protocol 1) are deemed unlikely to effectively inactivate hydrophilic SAS. Being aware that protein adsorption may be higher or different for hydrophobic SAS, we conclude that the uniform reduction of bioactivity found here for surface-treated SAS primarily relies on the capping/chemical modification of silanol groups with only a small, if any, supplementation due to limited BSA binding.

To further unravel the possible effects of Protocol 1 on SAS effects, we also subjected the hydrophilic SAS to this protocol. Generally, there was a rightward shift of the dose–response curves for LDH and GLU quantitatively reflected by increased EC_50_ values (Supplementary Figures S6, S7 and Table 4). We hypothesize that this mitigation of bioactivity may have been caused by a reduction of aggregate/agglomerate size which likely lowers particle settling and, therefore, reduces the availability of particles at the bottom of the culture dish. In fact, CAB-O-SIL® S17D and CAB-O-SIL® EL-90 showed a reduced HD of aggregates/agglomerates in F-12K medium. It is furthermore in line with this hypothesis that shifts of the EC_50_ values upon Protocol 1 were hardly measurable for LUDOX® TM-50 and LUDOX® SM, both of which are colloidal SAS whose single monodisperse particles were not degraded by increased USD energy. We cannot exclude that the combined ethanol/BSA pre-treatment of Protocol 1 contributes to the mitigation of the bioactivity of hydrophilic SAS. However, the missing effect of Protocol 1 on the bioactivity of LUDOX® SM argues against this assumption. A major difference between Protocol 1 and 2 concerned CAB-O-SIL® S17D: while Protocol 1 led to monophasic dose–response curves of CAB-O-SIL® S17D (Figure 3), biphasic dose–response curves were obtained upon protocol 2 for the release of LDH, GLU, and TNFα (Table 3). At least for TNFα, similar biphasic responses to SAS have been found earlier and have been interpreted as a rapid destruction of the cells and/or antigens due to elevated particle concentrations (37). Although this may apply also for enzymes such as LDH and glucuronidase, further analysis is needed to understand the biphasic effects of CAB-O-SIL® S17D. Overall, changes in particle size may account for the EC_50_ shift of untreated SAS upon Protocol 1.

In any case, it should be kept in mind that—regardless of the dispersion protocol used—non-treated but also the slightly hydrophilic surface-treated SAS AEROSIL® R 816 was far more bioactive than surface-treated highly hydrophobic SAS. Unlike untreated SAS, all surface-treated hydrophobic SASs were classified as passive materials (Supplementary Table S1), following the previously established evaluation criteria which consider the BET surface and the number of positive test results in the alveolar macrophage assay (34). While organosilane treatment can also dampen the bioactivity of crystalline silica (33, 49), we propose that the mechanism by which the *in vitro* bioactivity of SAS is ruled out not only involves the inactivation of silanol groups but also benefits from hydrophobic molecules at the particle surface.

## Data Availability Statement

The original contributions presented in the study are included in the article/Supplementary Material, further inquiries can be directed to the corresponding author/s.

## Author Contributions

MW and TS took a leading role in project administration and prepared the manuscript. AV was involved in toxicity testing, particle size measurements, and preparation of the manuscript. JN served as scientific advisor and prepared the manuscript. NK was involved in project planning, funding acquisition, and preparation of the manuscript. All authors contributed to the article and approved the submitted version.

## Funding

This work was funded by the SAS for REACH Consortium GbR for conducting of the work at IBE R&D Institute for Lung Health gGmbH.

## Conflict of Interest

MW and AV were employed by IBE R&D Institute for Lung Health gGmbH, a non-profit research institute, and received funding from the Evonik Operations GmbH for conducting the study and preparing the manuscript. TS and NK were employed by Evonik Operations GmbH. JN was employed by Grace Europe Holding GmbH.

## Publisher's Note

All claims expressed in this article are solely those of the authors and do not necessarily represent those of their affiliated organizations, or those of the publisher, the editors and the reviewers. Any product that may be evaluated in this article, or claim that may be made by its manufacturer, is not guaranteed or endorsed by the publisher.
