# Supplementary material for: Surface Treatment With Hydrophobic Coating Reagents (Organosilanes) Strongly Reduces the Bioactivity of Synthetic Amorphous Silica in vitro
Source: Front Public Health. 2022 Jun 21;10:902799. doi: 10.3389/fpubh.2022.902799 (PMC9253389; doi:10.3389/fpubh.2022.902799)
Supplement: Supplementary file 1 [file Data_Sheet_1.docx]

*Supplementary Information*

**Surface Treatment with Hydrophobic Coating Reagents (Organosilanes) Strongly Reduces the Bioactivity of Synthetic Amorphous Silica in vitro**

Martin Wiemann^1*^, Antje Vennemann^1^, Tobias B. Schuster^2^, Jürgen Nolde^3^

and Nils Krueger^2^

^1^ IBE R&D Institute for Lung Health gGmbH, Mendelstr. 11, D-48149 Münster, Germany; [martin.wiemann@ibe-ms.de](mailto:martin.wiemann@ibe-ms.de) (ORCID ID: 0000-0002-8230-8601); vennemann@ibe-ms.de

^2^ Evonik Operations GmbH, Rodenbacher Chaussee 4, 63457 Hanau-Wolfgang, Germany; [tobias.schuster@evonik.com](mailto:tobias.schuster@evonik.com) (ORCID ID: 0000-0003-3729-8683); [nils.krueger@evonik.com](mailto:nils.krueger@evonik.com)

^3^ Grace Europe Holding GmbH, Worms, Germany; Juergen.Nolde@grace.com (ORCID ID: 0000-0001-7084-7505)

* Corresponding Author:

Prof. Dr. Martin Wiemann

IBE R&D Institute for Lung Health gGmbH

Mendelstr. 11

48149 Münster

Germany

Phone +49-251-9802340

Fax +49-251-9802349

Email martin.wiemann@ibe-ms.de

CAB-O-SIL® S17D CAB-O-SIL® (E)L 90

**
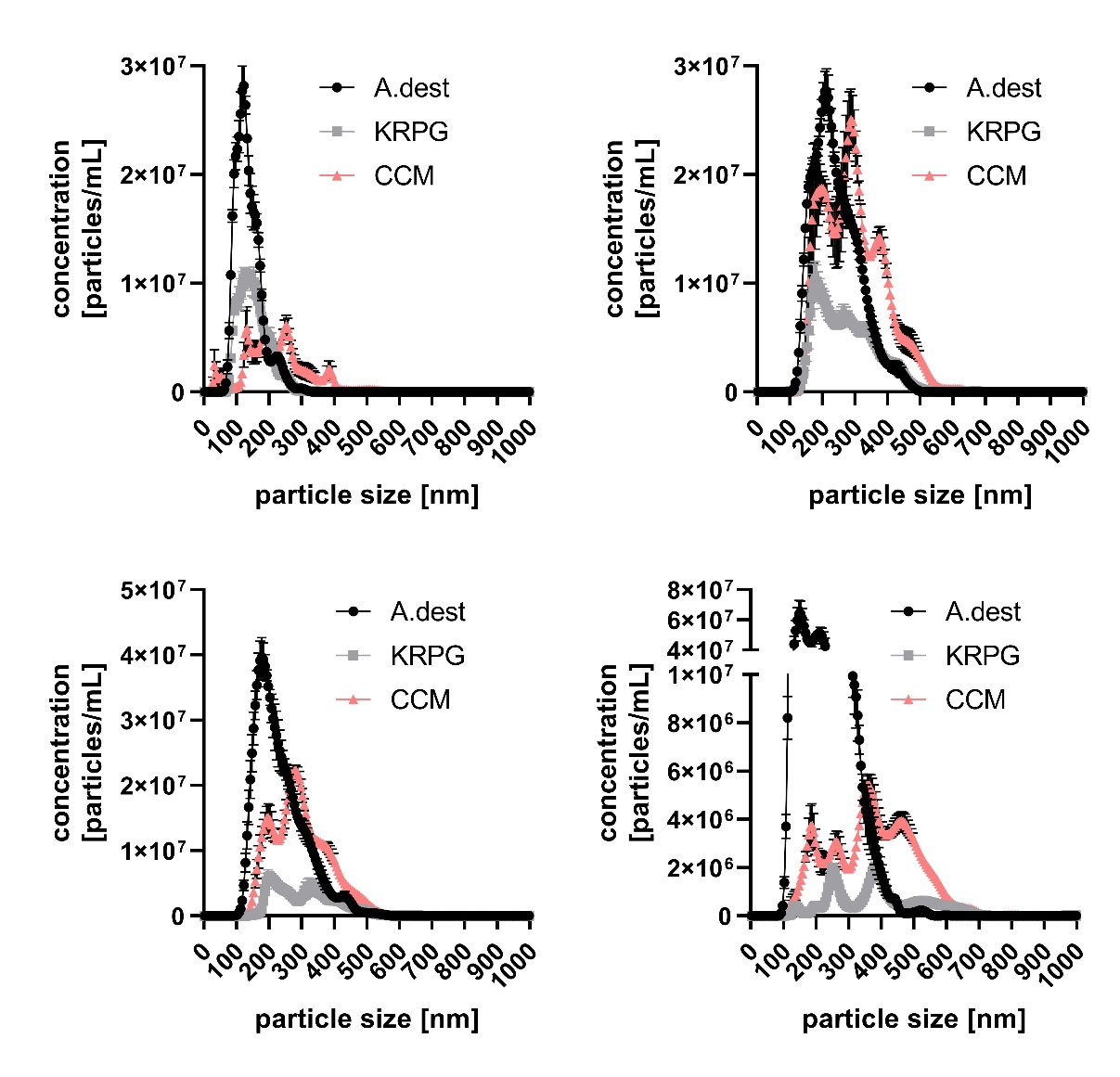
**

AEROSIL® 50 AEROSIL® R 816

**Figure S1.** Size distribution of three uncoated and one slightly hydrophilic surface treated SAS (AEROSIL® R816), all dispersed with 270 J/mL as analyzed by particle trackng analysis (PTA). Measurements were carried out in H_2_O (A. dest), KRPG buffer (KRPG), and F-12K medium (complete cell culture medium: CCM). The uncoated SAS LUDOX® SM and LUDOX® TM were not detectable and, therefore, not analyzed.


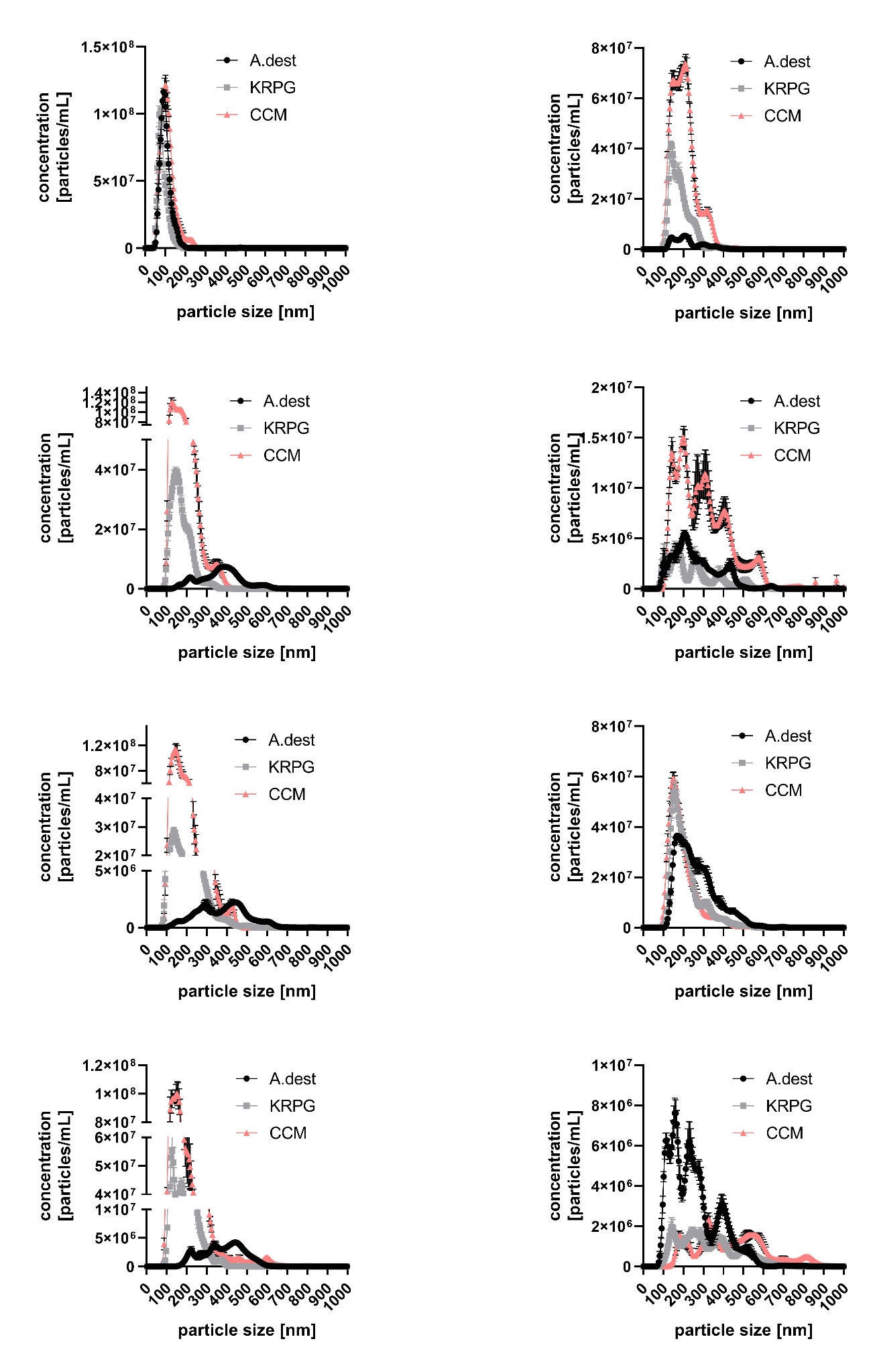


CAB-O-SIL® TGC413 HDK® H15

CAB-O-SIL® TS610 AEROSIL® R504

AEROSIL® R711 SIPERNAT® D17

CAB-O-SIL® TS720 HDK® H2000

**Figure S2.** Size distribution of hydrophobic surface treated SAS particles dispersed with the NanoGenoTox protocol, as analyzed by PTA. Measurements were carried out in H_2_O (A. dest), KRPG buffer (KRPG), and F-12K medium (complete cell culture medium: CCM).

**Tabelle S1**. Agglomeration and sedimentation behavior (Protocol 1) and biologic effect of SAS in the macrophage model (active/passive categorization).

**Substance name Change in Settled Bioactivity^3)^**

**Agglomerate Agglomerates**

**Size (PTA)^1)^ visible^2)^**

AEROSIL® R816 +21.5% (+101%) ++ passive

AEROSIL® R504 +51% ++ passive

CAB-O-SIL® TGC413TRD +8.1% + passive

CAB-O-SIL® TS720 +58.4% + passive

HDK® H15 +8.4% - passive

HDK® H2000 +164.6% ++ passive

AEROSIL® R711 - 63.7% + passive

SIPERNAT® D17 - 12.6% ++ passive

CAB-O-SIL® TS610 - 66.9% (+) passive

CAB-O-SIL® S17D +14.5% (+30.4%) (+) active

CAB-O-SIL® (E)L-90 +25.4% (+10.9%) + active

LUDOX® SM n.m. (n.m) - passive

LUDOX® TM-50 n.m. (n.m) - active

AEROSIL® 50 +54.1% (42.4%) (+) active

**Footnotes to Table S1:**

^1)^ Numbers give the relative increase in hydrodynamic diameter (mode values taken from Table 2 of the Main Manuscript) upon transfer from aqueous dispersion (prepared according to Protocol 1) to F-12K medium. Final particle concentration amounted to f 90 µg/mL. Numbers in brackets are based on aqueous suspension prepared according to Protocol 2.

^2)^ Particles were viewed with phase contrast microscopy using a 20x objective mounted to a Nikon Biostation.

^3)^ According to Wiemann et al. 2016 (see Reference List of the main manuscript)

n.m.: not measurable with particle tracking analysis (PTA)

PTA: particle tracking analysis

- : no sediments, (+): few scattered aggregates/agglomerates, + sparse layer of aggregates/ agglomerates, ++: dense layer of aggregates/agglomerates


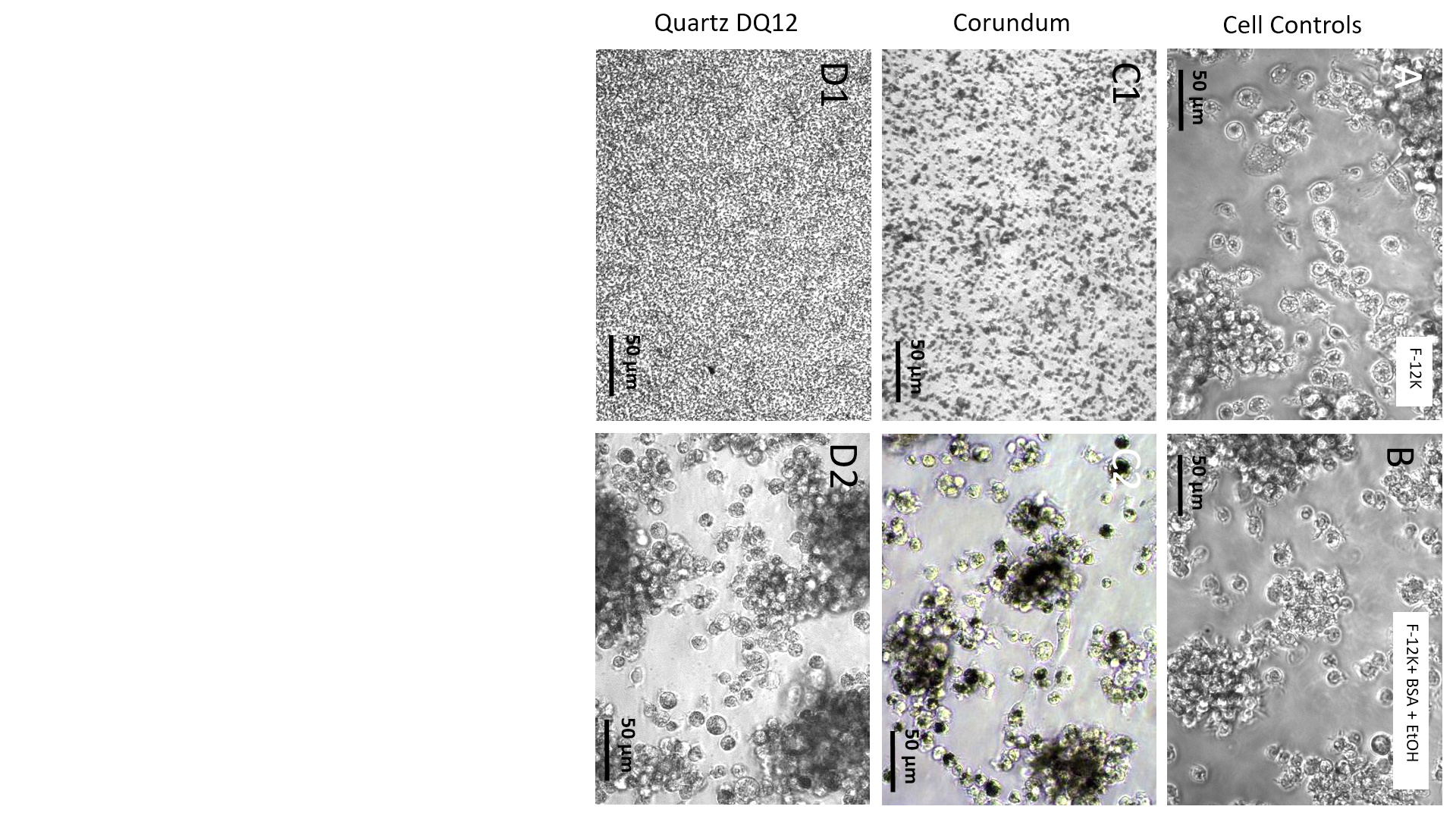


**Figure S3.** *Upper row:* Light microscopy of cell controls in F-12K medium, and in F-12K medium with trace amounts of ethanol and BSA as used for the NanoGenoTox protocol. *Middle row*: gravitationally settled corundum (180 µg/mL, left) and NR8383 cells laden with the same amount of particles (right). Lower row: gravitationally settled quartz DQ12 (180 µg/mL, left) and NR8383 cells laden with the same amount of particles (right).


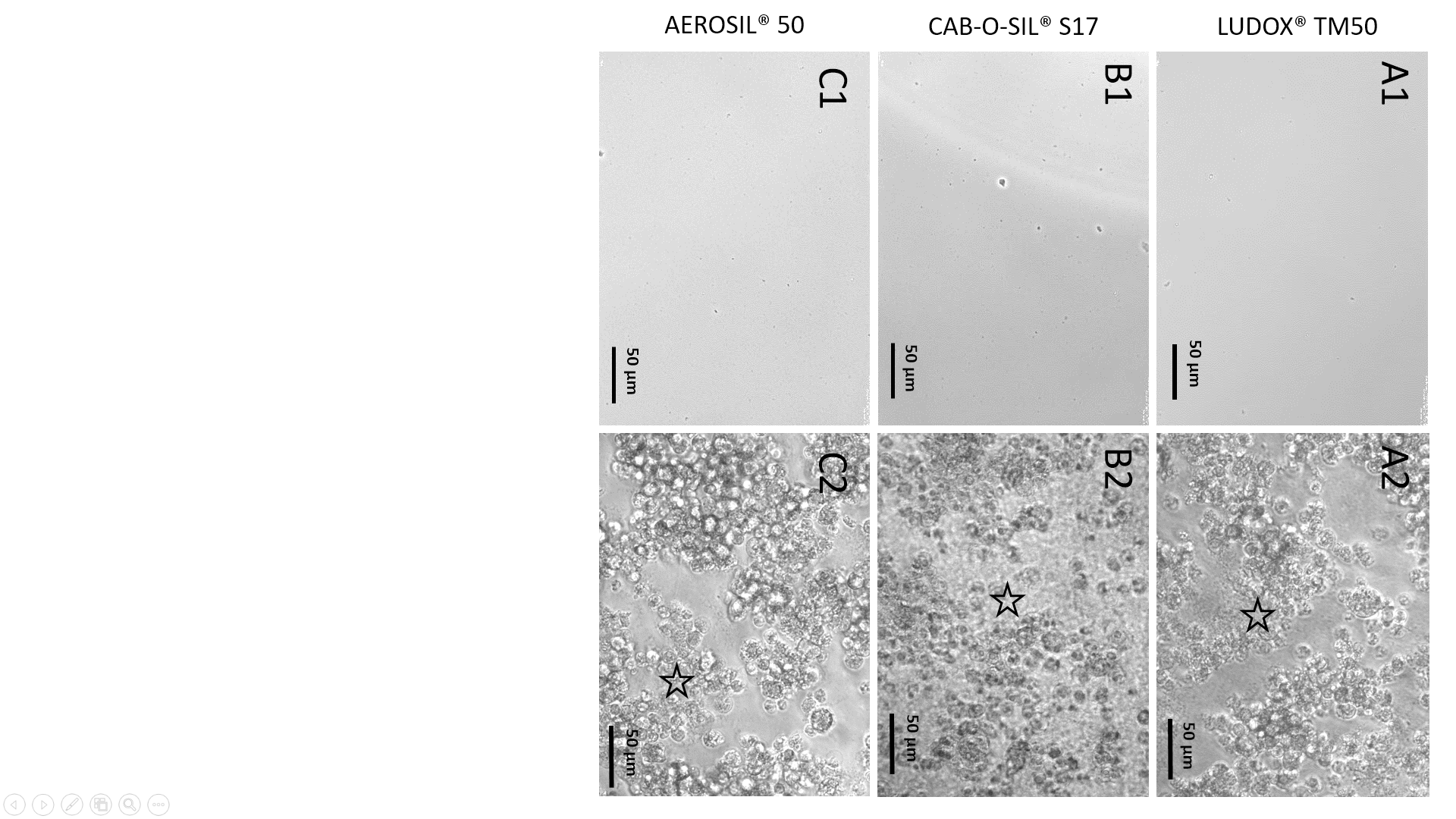


**Figure S4.** Phase contrast images of NR8383 cells after treatment with untreated SAS. *Left:* Light microscopy of gravitationally settled LUDOX® TM50, CAB-O-SIL® S17, and AEROSIL® 50 (90 µg/mL). Particles were dispersed with Protocol 2 and sediments were viewed after 16 h of incubation under cell culture conditions. Apart from a few scattered aggregates no settled matter is visible. *Right:* NR8383 macrophages exposed to the same amount of particles for 16 h. Necrotic cells have a pale and/or granular appearance and irregular, deteriorated contours (asterisks). This is especially obvious after administration of CAB-O-SIL® S17.


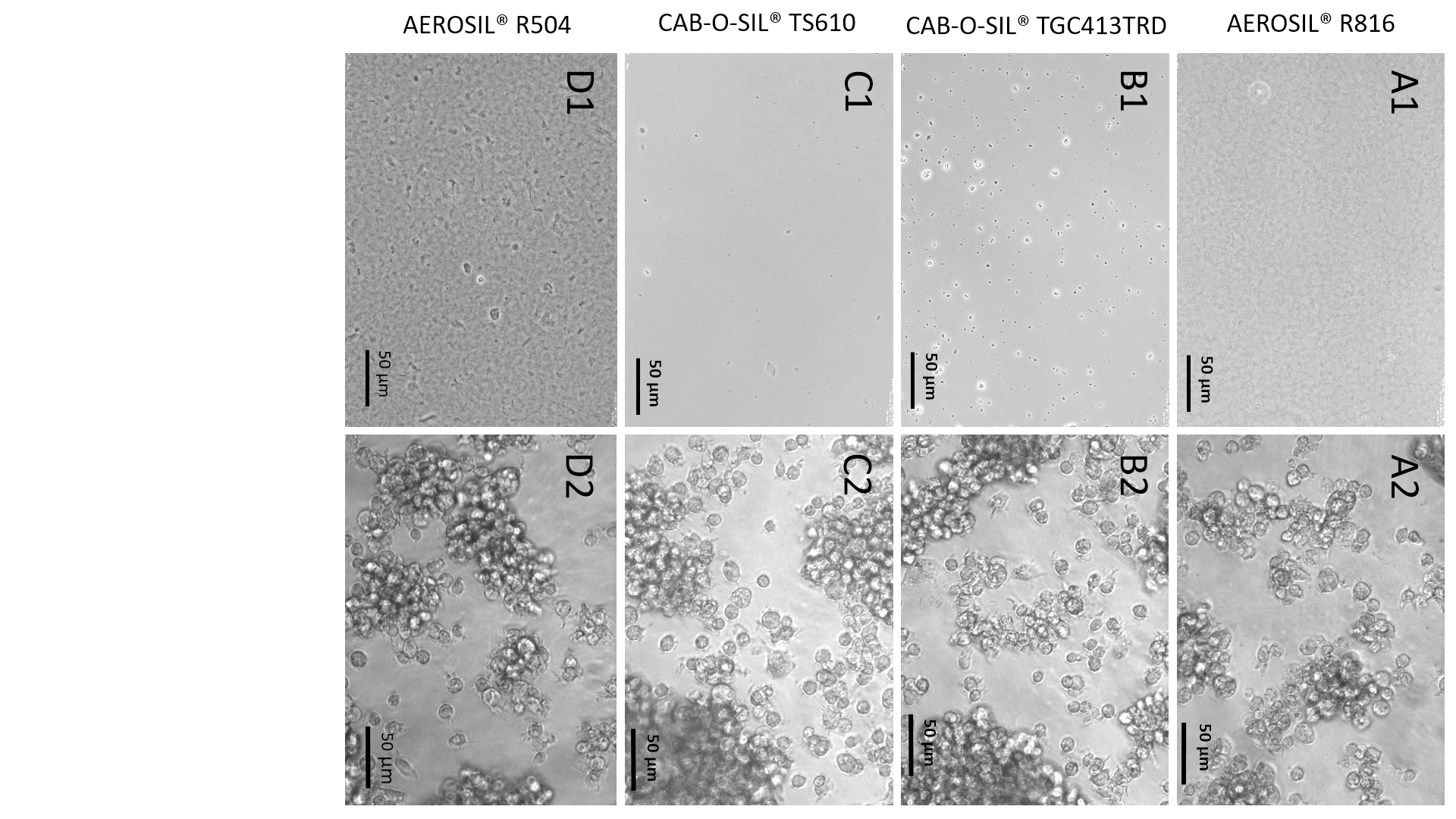


**Figure S5.** Phase contrast images of NR8383 cells after treatment with surface-treated hydrophobic SAS. *Left:* Light microscopy of gravitationally settled AEROSIL® R816, CAB-O-SIL® TGC413TRD, CAB-O-SIL® TS 610, and AEROSIL® R504 (90 µg/mL). Particles were dispersed with Protocol 1 (except for AEROSIL® 816 which was dispersed with Protocol 2) and sediments were viewed after 16 h of incubation under cell culture conditions. Settled agglomerates are visible for AEROSIL® R816 and and AEROSIL® R504. *Right:* NR8383 macrophages exposed to the same amount of particles for 16 h. no necrotic cells are visible.


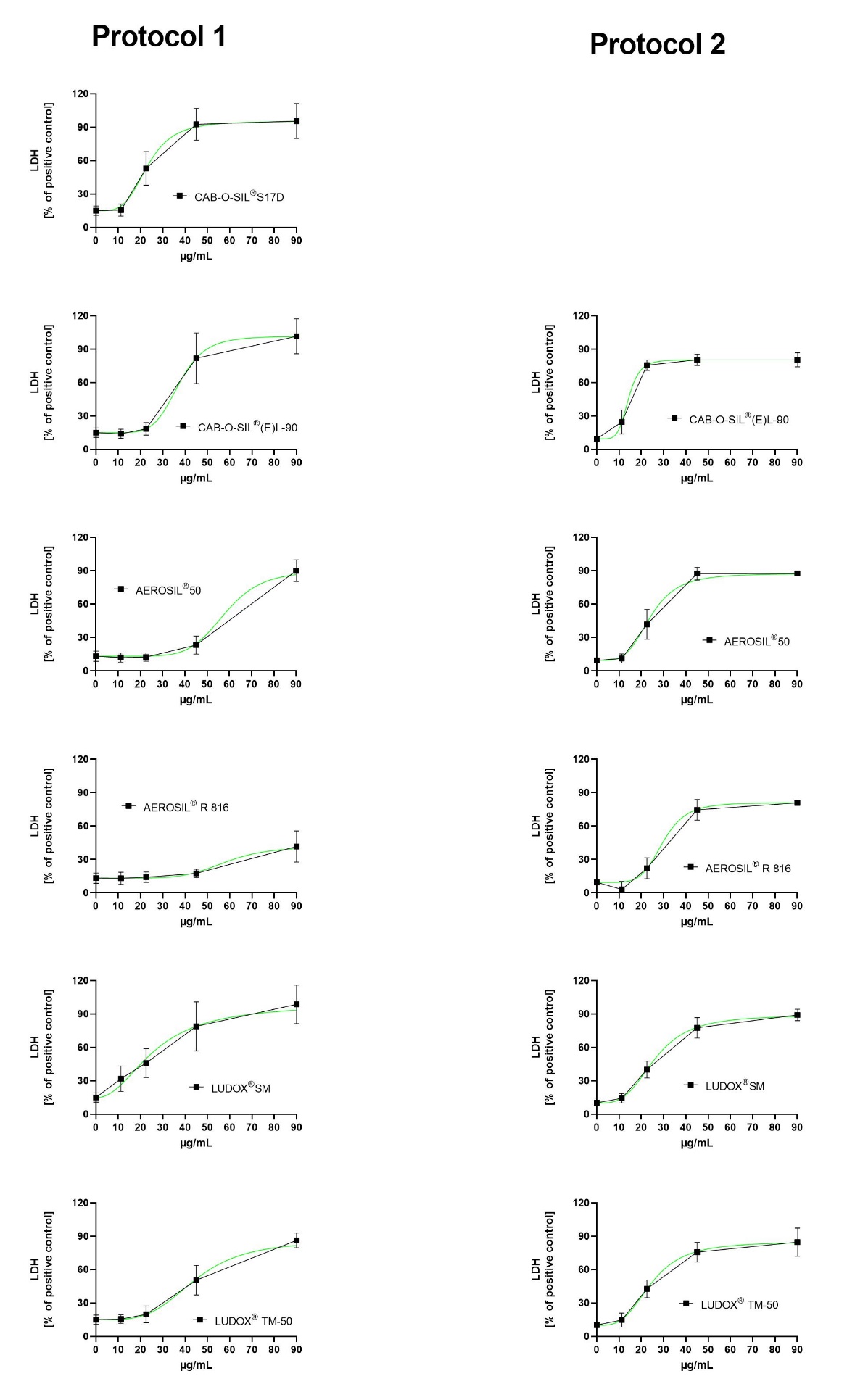


**Figure S6.** Dose-response curves for lactate dehydrogenase (LDH) release after treatment of NR8383 cells with increasing concentrations of hydrophilic SAS dispersed with Protocol 1 (left) or Protocol 2 (right) . Fitted curves (green) were used for calculating EC_50_ values.


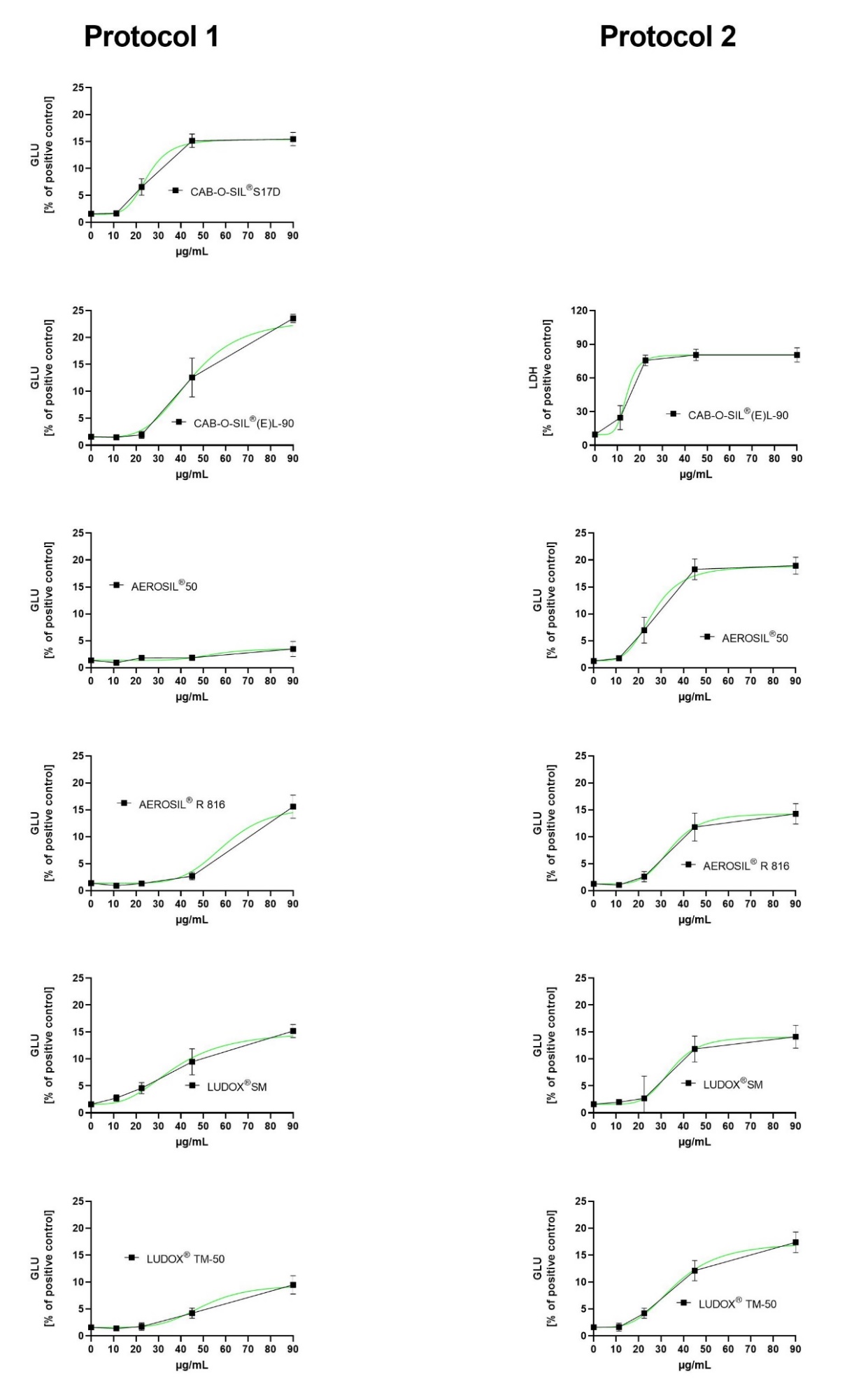


**Figure S7.** Dose-response curves for glucuronidase (GLU) release after treatment of NR8383 cells with increasing concentrations of hydrophilic SAS dispersed with Protocol 1 (left) or Protocol 2 (right) . Fitted curves were used for calculating EC_50_ values.


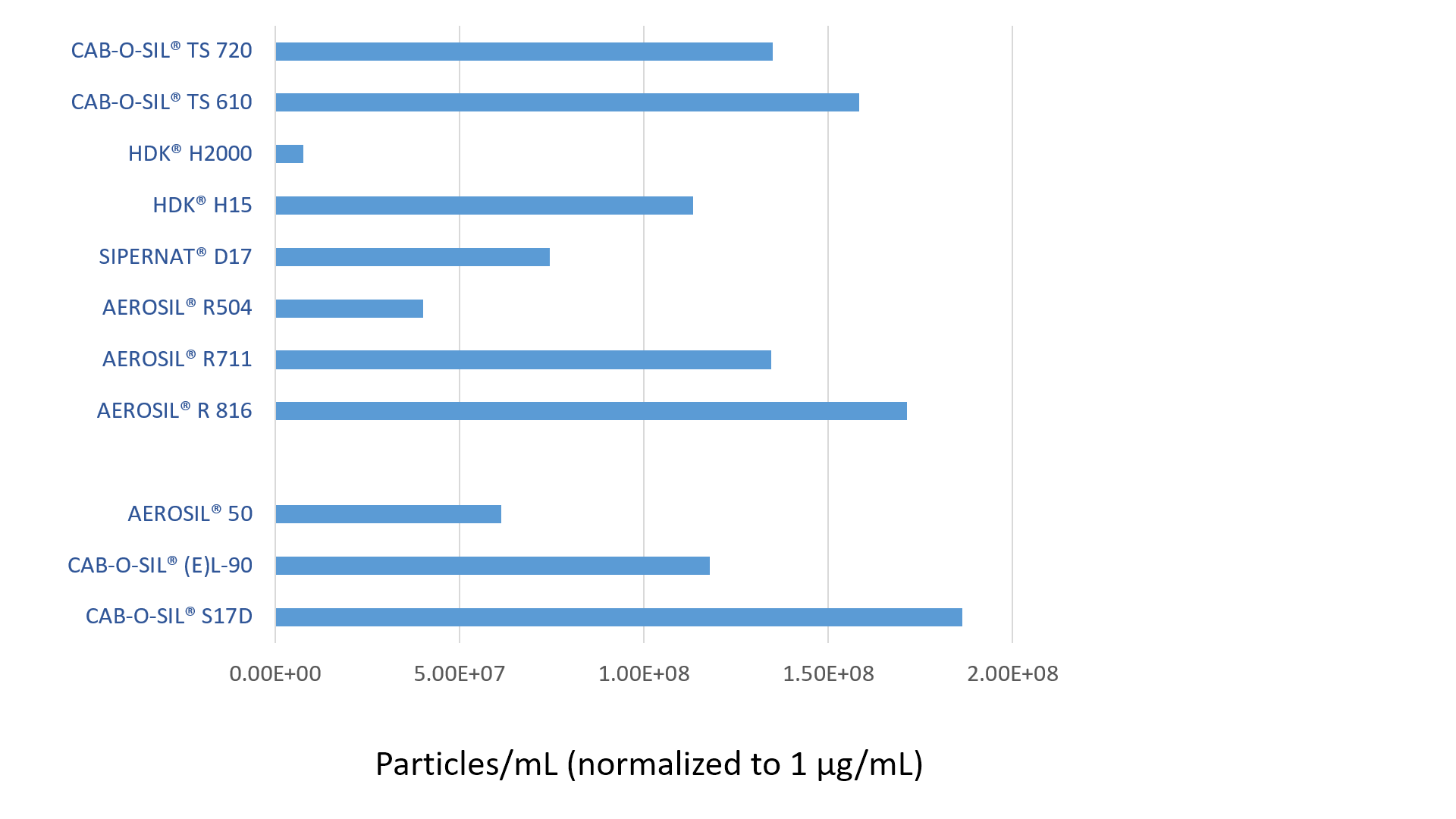


**Figure S8.** Concentration of small particles (hydrodynamic diameter <0.5 µm) in F-12K medium as dispersed with Protocol 1. Concentrations were taken from PTA measurement and reflect a single concentration approach (n=3). Because particle concentration needs to be optimized for optical tracking and varied in the measured suspensions from 0.18-18 µg/mL (see: Maguire et al. (2017) Benchmark of Nanoparticle Tracking Analysis on Measuring Nanoparticle Sizing and Concentration, https://doi.org/10.1115/1.4037124), all values were normalized to a concentration of 1 µg/mL.
